# Interactively addressable organic metadevices

**DOI:** 10.1038/s41467-026-75757-4

**Published:** 2026-07-16

**Authors:** Xiangyu Huang, Benjamin Renz, Yueqiang Hu, Na Liu

**Affiliations:** 1 2nd Physics Institute, University of Stuttgart, Stuttgart, Germany; 2 Max Planck Institute for Solid State Research, Stuttgart, Germany; 3 College of Mechanical and Vehicle Engineering, Hunan University, Changsha, P.R. China

**Keywords:** Optical materials and structures, Nanoscale devices, Materials for optics

## Abstract

Active, pixel-level addressability is central to the realization of metadevices with programmable wavefront control. However, two-dimensional operation at visible wavelengths remains challenging, as reducing metasurface pixel dimensions imposes increasing constraints on electrical interconnect design and local tunability. Here, we demonstrate interactively addressable organic metadevices, in which ultrathin polyaniline is conformally integrated with plasmonic nanoantennas to yield individually switchable metasurface pixels with localized electrochemical modulation. Using a planar fan-out architecture, we realize two-dimensional metasurface arrays, in which each pixel is electronically isolated and driven at sub-volt voltages, enabling millisecond-scale switching dynamics. Embedded within a user-driven electronic control loop, the platform converts user inputs into pixel-level voltage patterns to generate reconfigurable holographic projections, supporting functionalities ranging from alphanumeric character rendering to interactive gaming. Pixel-resolved measurements reveal uniform electrochemical behaviour, negligible electrical crosstalk, and stable operation across dynamically evolving holographic scenes. Our results establish organic metasurfaces as a promising route toward user-programmable and interactively addressable photonic systems.

## Abstract (web summary)

An interactively addressable organic metadevice enables independent electrical control of metasurface pixels to generate reconfigurable holographic projections, providing a platform for programmable and user-interactive photonic systems.

## Introduction

Metasurfaces have become a versatile photonic platform, enabling ultrathin devices that manipulate the amplitude, phase, and polarization of light with subwavelength spatial resolution^1–5^. As optical applications increasingly demand programmability and dynamic control, the field is transitioning from static elements toward actively addressable metadevices^5–22^. A broad range of tuning strategies have been explored to introduce dynamic functionality. For instance, in the visible spectral range Kuznetsov and coworkers demonstrated one-dimensional (1D) metasurface-based spatial light modulators (SLMs) employing liquid crystals (LCs)^10–12^. In the near-infrared (NIR), Park et al. realized an all-solid-state active metasurface based on dual-gated field-effect modulation of indium tin oxide (ITO)^13^, while Shirmanesh et al. demonstrated a reconfigurable metasurface using 1D electrical addressing^15^. These studies underscore the importance of jointly engineering photonic architectures with their driving electronics. However, 1D routing schemes inherently limit the number and scalability of independently addressable pixels, thereby constraining the spatial degrees of freedom required for complex light control.

To fully exploit the potential of active optical metasurfaces, two-dimensional (2D) electrical addressing with independent pixel-level control is essential^5,6^. Such 2D addressing has been demonstrated in the NIR for beam steering^14^, and extended to the visible through integration of LCs^19^. Alternative strategies employing external modulation platforms, such as SLMs and digital micromirror devices, can impart dynamic functionality to static metasurfaces^23–27^. A recent work by Fan et al. demonstrated an optically addressed metasurface SLM^28^. While powerful, these hybrid systems are not intrinsically active optical devices, limiting compactness and on-chip integration. Recently, organic conducting polymers (CPs) have emerged as a promising material platform for reconfigurable metasurfaces^29–50^. Their electrochemical response, governed by voltage-controlled redox chemistry, enables large and reversible modulation of the refractive index and absorption at low operating voltages^7,29,49–55^. Crucially, CPs can be conformally grown as ultrathin coatings around nanoscale photonic structures, and their modulation does not rely on collective molecular reorientation, mitigating pixel crosstalk common to LC-based tuning^6,18,19,29–31^. Although CP-integrated metasurfaces have enabled dynamic beam control with multiple independently driven channels, previous implementations have largely been restricted to the NIR^35,56^ or relied on 1D electrical addressing^30^. As a result, high-density, fully addressable 2D metasurface architectures operating at visible wavelengths remain largely unexplored.

Here, we demonstrate 2D, interactively addressable organic metadevices operating at visible wavelengths. By locally integrating ultrathin polyaniline (PANI) with plasmonic nanoantennas^29–31^, we realize individually switchable electro-optic metasurface pixels arranged in 2D arrays. Each pixel is independently addressable through a planar ITO fan-out network, simplifying electrical routing and facilitating device integration. The metadevice exhibits millisecond-scale switching dynamics at sub-volt operating voltages, together with negligible electrical crosstalk and uniform electrochemical behavior across the array. Integrated into a closed electronic feedback loop, the platform translates user inputs into pixel-level driving signals, enabling real-time interactive holographic operation. Leveraging this architecture, we demonstrate system-level programmability, exemplified by live character rendering and interactive holographic gaming. This work establishes a promising route toward fully addressable 2D organic meta-optics, paving the way for compact, user-interactive photonic systems.

## Results

### Design and implementation of the metadevice

**Fig. 1:**
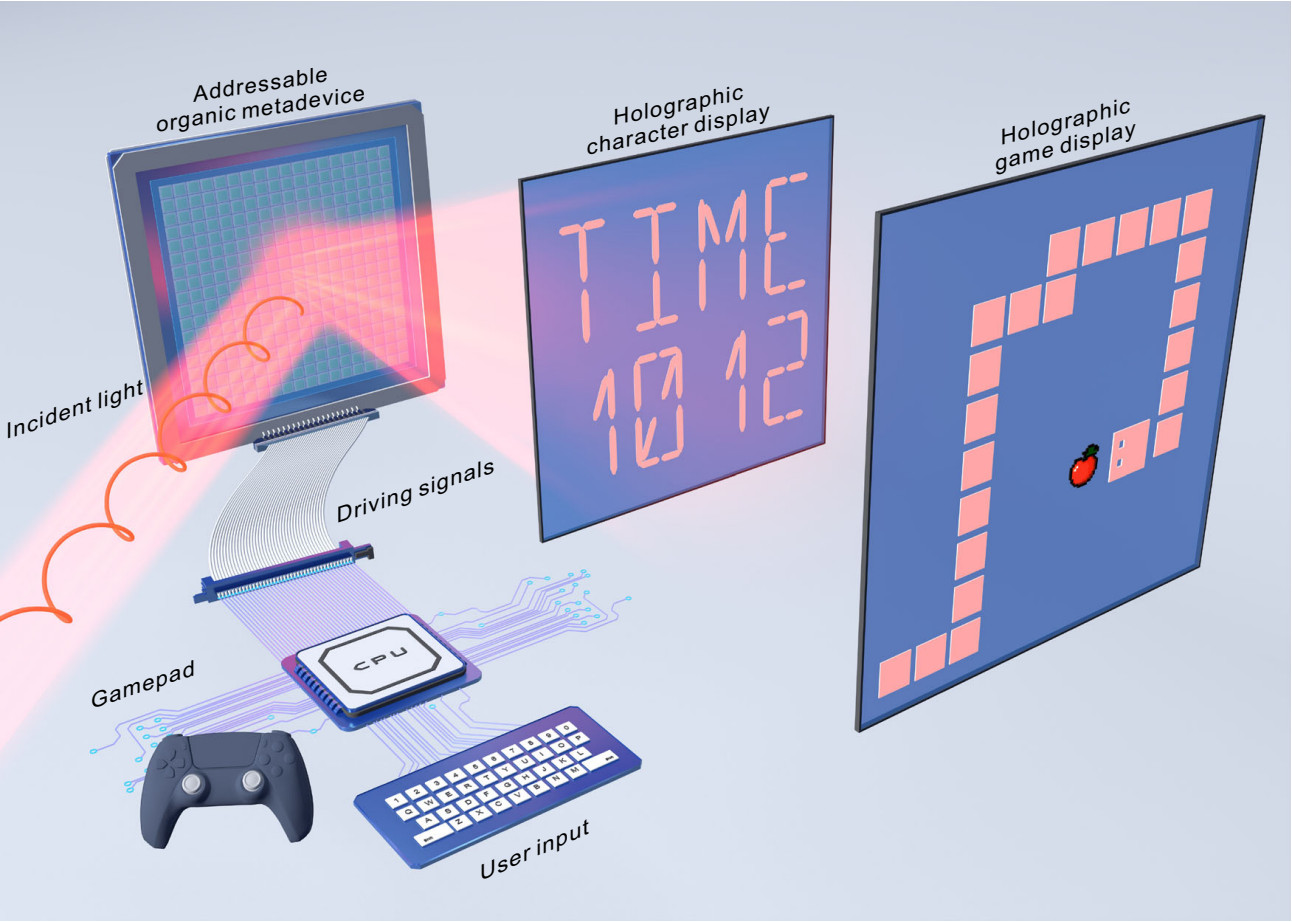
Schematic illustration of a 2D interactively addressable metadevice. The device comprises an array of electrically addressable metasurface pixels, each containing gold nanorods (AuNRs) that encode a predefined holographic phase profile. The pixels modulate the wavefront of reflected light to reconstruct holographic images in the far field. The AuNRs are conformally coated with an ultrathin conducting polymer layer, enabling electrochemical switching of individual pixels between on/off states. The metadevice operates within a closed electronic loop, in which user inputs from interactive interfaces, such as a keyboard for character rendering or a gamepad for interactive gaming, are processed by control electronics and converted into pixel-level electrical driving signals, allowing dynamic holographic projections.

Figure 1 illustrates the schematic of the 2D interactively addressable metadevice. It comprises an array of metasurface pixels, each controlled by an individual electrode to support user-interactive operation. Each pixel contains a periodic arrangement of gold nanorods (AuNRs) that encode the desired holographic phase profile through their Pancharatnam–Berry (PB) phase orientations^57–60^, collectively shaping the reflected wavefront. The AuNRs are conformally coated with an ultrathin PANI layer, whose voltage-dependent electrochemical switching enables localized electro-optic modulation. The metadevice operates within a closed electronic loop, where user inputs from peripherals, such as a keyboard or gamepad, are processed by a computer-based control interface and converted into pixel-level driving signals for frame-by-frame holographic updates.

**Fig. 2:**
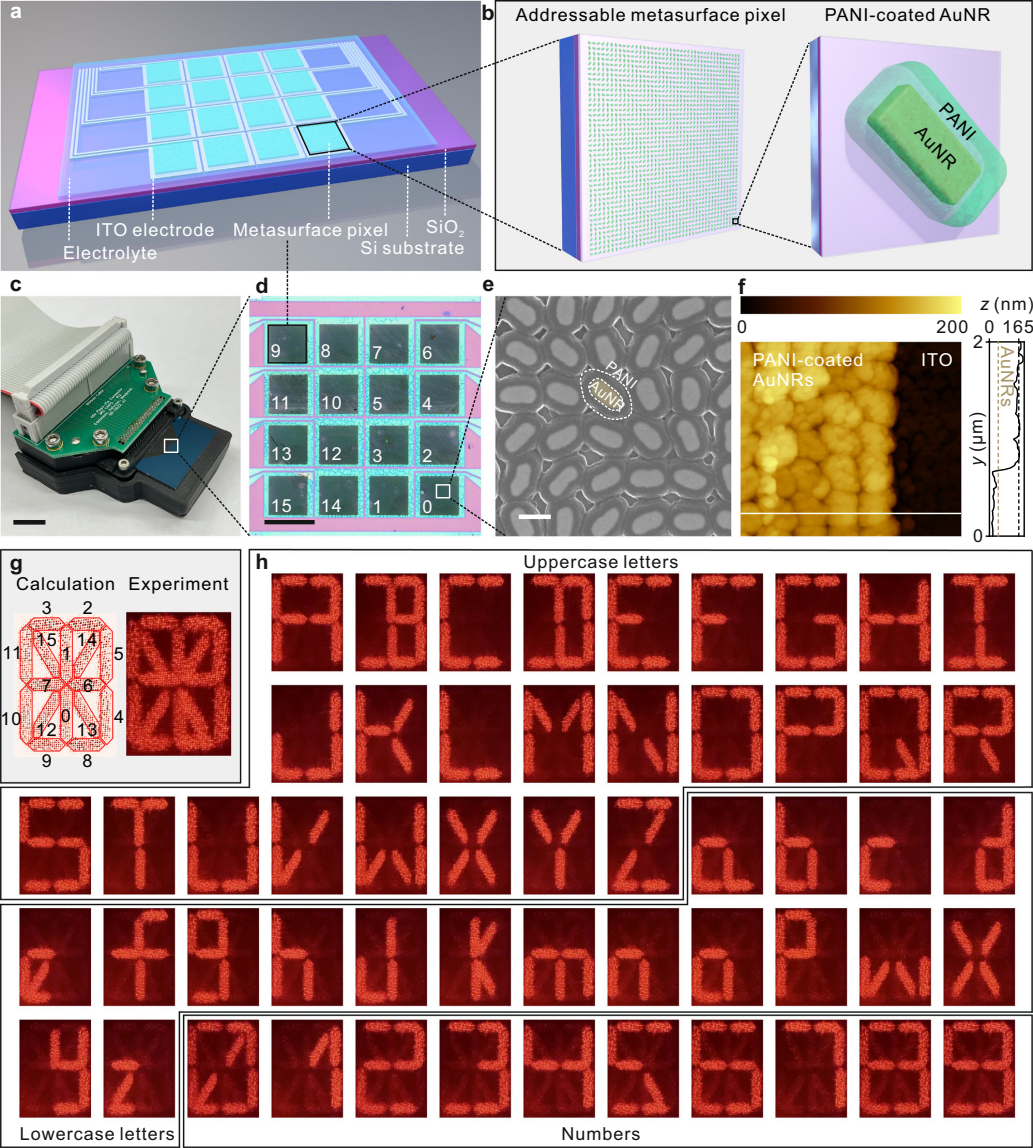
Design and characterization of an organic metadevice with a 4 × 4 array of metasurface pixels. **a** Device-level schematic of the organic metadevice, comprising a 4 × 4 array of electrically isolated metasurface pixels fabricated on a reflective Si/SiO_2_ substrate and connected to individual indium tin oxide (ITO) fan-out electrodes for independent electrical addressing. **b** Zoomed-in schematic illustrating a single metasurface pixel (left) and an individual polyaniline (PANI)-coated AuNR (right). **c** Photograph of the fabricated metadevice mounted on a custom sample holder and interfaced with external electrical connections. Scale bar, 1 cm. **d** Optical microscopy image of the metasurface array. Scale bar, 350 μm. **e** SEM image showing conformal PANI coating around individual AuNRs within a metasurface pixel. Scale bar, 200 nm. **f** AFM topography of the metasurface pixel, with the height profile extracted along the white line, revealing the vertical thickness of the PANI shell. **g** Calculated far-field holographic image generated by the 16-segment metasurface (left) and the corresponding experimentally reconstructed hologram (right), illustrating the one-to-one mapping between individual pixels and holographic segments. **h** Representative experimentally reconstructed holograms forming a look-up table of alphanumeric characters, including uppercase and lowercase letters and numbers.

Figure 2a shows the layout of a representative metadevice comprising a 4 × 4 array of metasurface pixels. Detailed fabrication procedures are provided in Supplementary Note 1 and Supplementary Fig. 1. Each pixel (350 μm pitch) contains AuNRs (220 nm × 110 nm × 50 nm) arranged in 300 nm × 300 nm unit cells (Fig. 2b). The AuNRs are oriented to implement an eight-level PB phase profile and are coated with a PANI layer deposited via electrochemical polymerization. Independent electrical addressing is achieved by routing each pixel to an individual ITO fan-out electrode, which interfaces directly with the sample-holder pins (Fig. 2c), ensuring fully independent pixel control across the array. Figure 2d presents an optical microscopy image of the fabricated metadevice, revealing uniform PANI polymerization across all pixels. The scanning electron microscopy (SEM) image confirms localized, conformal PANI growth around individual AuNRs, forming shell-like coatings (Fig. 2e). Atomic force microscopy (AFM) characterization reveals a total structural height of ~165 nm (Fig. 2f). Given the 50 nm height of the AuNRs, the vertical PANI thickness is therefore ~115 nm, exceeding the lateral shell thickness of ~75 nm measured by SEM. This anisotropic growth is likely attributed to the larger free volume available above the AuNRs compared with the in-plane surroundings. Finite-element simulations elucidating the dependence of anomalous reflection intensity on AuNR geometry and electrochemical state of PANI are presented in Supplementary Note 2 and Supplementary Figs. 2, 3. The binary on/off behavior of each metasurface pixel arises from the electrochemical modulation of the PANI shell. Under applied bias, PANI undergoes reversible redox transitions between a reduced, low-absorption state and an oxidized, high-absorption state, accompanied by pronounced changes in the complex refractive index^61^. These changes detune the plasmonic resonance of the underlying AuNRs and modulate their scattering amplitude^29^. The encoded PB phase profile is retained in the reduced and oxidized PANI states (Supplementary Fig. 4). In the reduced state, the PANI-coated AuNRs exhibit pronounced plasmonic scattering, whereas in the oxidized state, increased absorption suppresses scattering (Supplementary Fig. 6). This ultrathin, conformal PANI coating is therefore critical for achieving fast, spatially localized, and high-contrast electro-optic modulation.

Each metasurface pixel generates a switchable holographic pattern, which corresponds to a segment of an alphanumeric symbol in the calculated far-field image (Fig. 2g). The angular distribution of the 16 segments is provided in Supplementary Fig. 7. Experimentally reconstructed holograms are projected onto a screen and captured using a visible-light camera (Fig. 2h; Supplementary Fig. 8). These holograms form a look-up table comprising 62 alphanumeric characters, including uppercase and lowercase letters (A–Z, a–z), and numbers (0–9). Each character corresponds to a 16-bit voltage vector *V*_character_ that specifies the on/off state of the 16 pixels (Supplementary Table 1). Iterating through this look-up table enables sequential holographic rendering of all encoded characters.

### Interactive holographic character display

**Fig. 3:**
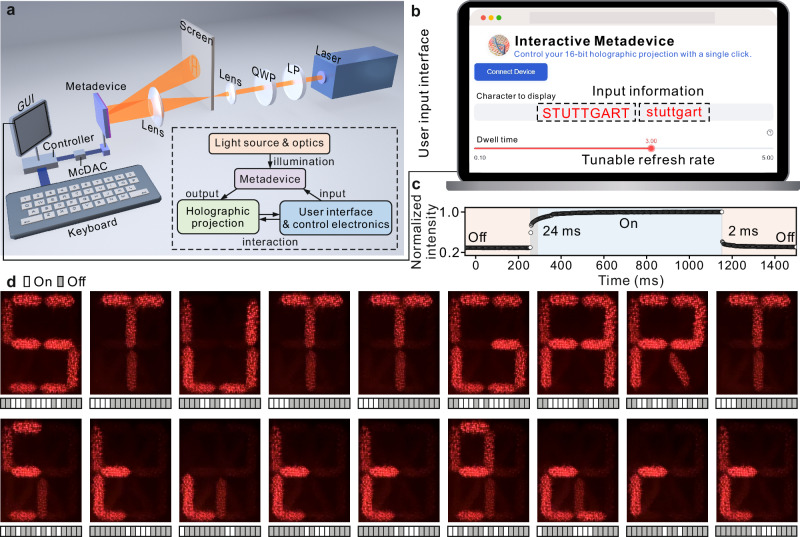
Interactive holographic character display. **a** Schematic of the optical setup and electronic control system used for interactive holographic projection. User inputs are processed through a graphical user interface (GUI) and converted into pixel-level driving signals via a multichannel digital-to-analog converter (McDAC). The inset shows a block diagram of the closed-loop system. **b** Screenshot of the GUI, allowing users to connect the metadevice, input character strings, and set programmable dwell times. **c** Temporal response of a representative metasurface pixel under voltage modulation between −0.2 V (on) and +0.6 V (off), showing the switching dynamics. **d** Experimentally reconstructed holographic projections of the input strings STUTTGART and stuttgart at a user-defined dwell time. The corresponding pixel on/off configurations for each frame are shown below the holograms.

To evaluate the interactive functionality of the metadevice, we implement a holographic projection system. As illustrated in Fig. 3a, a collimated 633 nm laser is converted to circularly polarized light (CPL) using a linear polarizer (LP) and a quarter-wave plate (QWP). The light beam is subsequently expanded by a pair of lenses to ensure uniform illumination of the 4 × 4 metasurface array. Upon reflection, the metasurface reconstructs off-axis holographic images on a distant screen. The metasurface chip is housed in an electrochemical cell filled with 0.5 M HNO_3_, which provides the ionic environment required for PANI redox switching. Electronic addressing is implemented through ITO fan-out electrodes connected to a multichannel digital-to-analog converter (McDAC) via a custom printed circuit board (PCB). System operation is coordinated by a laptop running a graphical user interface (GUI), which allows users to input character strings with programmable dwell times (Fig. 3b and Supplementary Movie 1). For each input, the corresponding 16-bit voltage vector *V*_character_ is retrieved from the look-up table (Supplementary Table 1) and applied to the metadevice, refreshing the holographic output accordingly.

To quantify the modulation performance, pixel switching dynamics are measured under voltage cycling between −0.2 V (on) and +0.6 V (off). The pixels exhibit rapid transitions, with switching times of 24 ms for the off-to-on transition and 2 ms for the reverse process, yielding an on/off intensity contrast of 3.5:1 (Fig. 3c). The intensity contrast can be improved by increasing the PANI thickness, with an accompanying trade-off in switching speed (Supplementary Fig. 10). These switching speeds correspond to modulation rates on the order of tens of hertz, sufficient for video-rate operation^34,62^. Figure 3d showcases representative holographic projections of the input strings STUTTGART and stuttgart (Supplementary Movie 1), demonstrating stable operation during programmed updates. The corresponding pixel activation patterns are shown alongside the reconstructed holograms.

### Interactive holographic game display

**Fig. 4:**
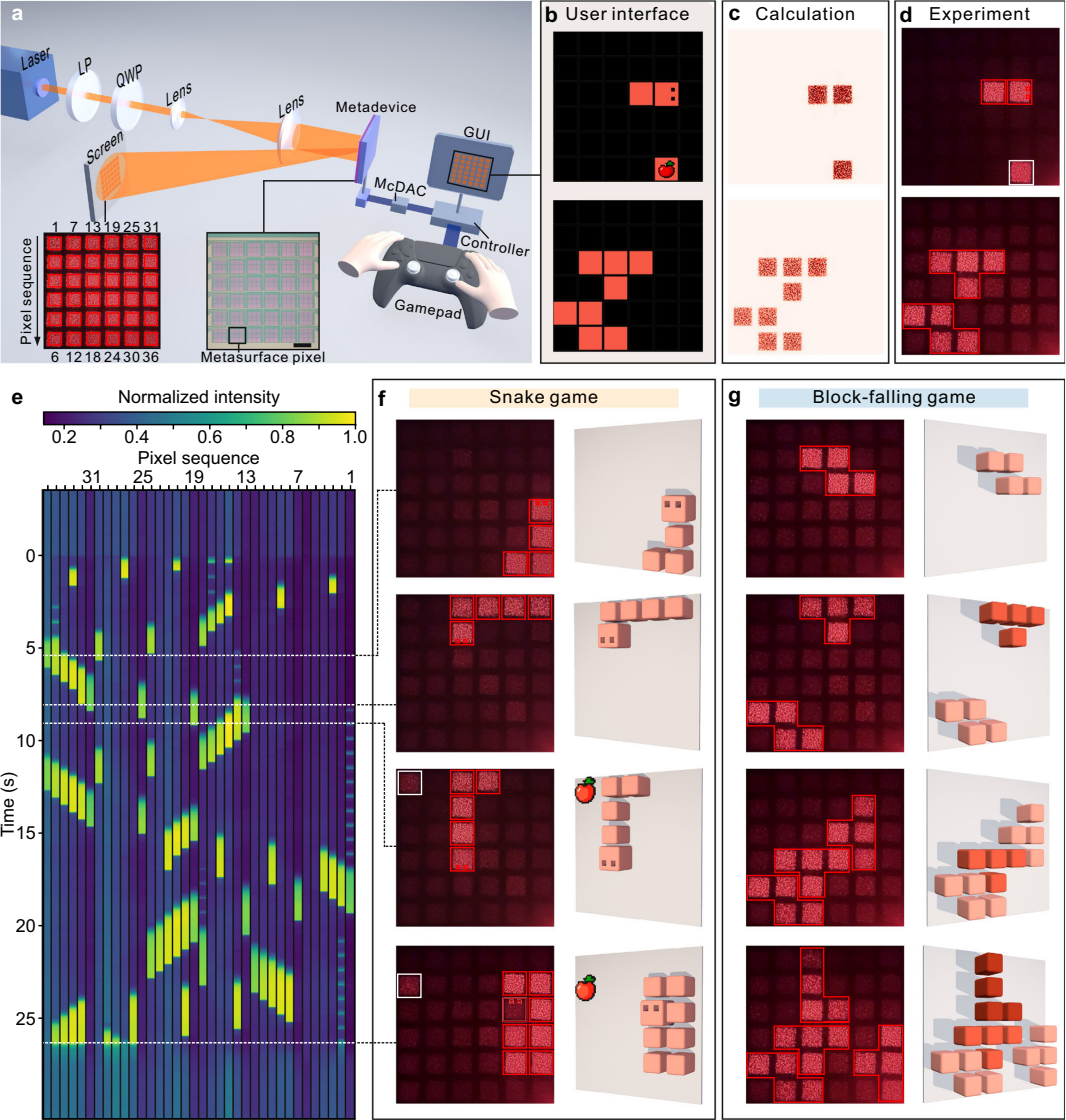
Interactive holographic game display based on a 6 × 6 addressable metadevice. **a** Schematic illustration of the optical measurement setup and electronic control architecture for interactive gameplay. A commercial gamepad provides user input, which is processed by the control electronics and translated into pixel-level driving signals for the metadevice. Insets show an experimentally reconstructed hologram with all 36 pixels in the on state, where each pixel region is marked by a red square and labeled with the pixel index used for analysis (left), and an optical microscopy image of the fabricated metadevice (right). Scale bar, 350 μm. **b** Representative GUI frames for the snake game (top) and the Tetris-like block-falling game (bottom). **c**, **d** Calculated far-field holographic patterns (**c**) and corresponding experimentally reconstructed holograms (**d**) for the pixel activation states shown in (**b**). For the snake game, the projected snake region is outlined in red, with the head-eye features indicated by red boxes, while the projected apple is outlined in white. For the block-falling game, the projected blocks are outlined in red. **e** Spatiotemporal intensity evolution of all 36 pixels (indexed as in (**a**)) during a representative snake game session. **f** Experimentally reconstructed holograms selected from the temporal sequence in (**e**) (corresponding to the white dashed lines), illustrating snake motion, snake growth, apple blinking, and the game-over state triggered by self-collision. Corresponding schematic illustrations of the game states are shown on the right. **g** Representative holographic frames of the Tetris-like block-falling game, showing user-controlled lateral shifting, rotation, and the game-over configuration (left), together with schematic illustrations of the corresponding game states (right).

To further demonstrate the versatility of the system, we implement a metadevice comprising 36 independently addressable metasurface pixels arranged in a 6 × 6 array (Fig. 4a). The device generates a holographically projected pixel array that serves as an interactive game field, spanning ~50 mm × 50 mm on a screen positioned 100 mm from the metadevice (Supplementary Note 3 and Supplementary Fig. 12). Pixel-resolved visibility evaluation of the holographically projected pixel array reveals clear contrast relative to the background and largely uniform optical performance across the array (Supplementary Note 3; Supplementary Fig. 12). By sequentially switching on one pixel while keeping all other pixels in the off state, the addressed pixel exhibits much larger intensity variation than the non-addressed neighboring pixels, confirming negligible crosstalk in the metadevice (Supplementary Fig. 13). A commercial gamepad serves as the user input device and communicates with both the control laptop and the McDAC. The holographically projected field provides the visual interface for user interaction, while the GUI displays the evolving game logic together with the corresponding pixel activation states in real time. For each game state, the control system generates a 36-element voltage vector *V*_logic_, assigning −0.2 V and +0.6 V to switch individual pixels on and off, respectively. This vector is then converted into DAC output commands *V*_DAC_ to refresh all pixels simultaneously. The applied voltage patterns are mirrored in the GUI for real-time monitoring. Representative GUI views for the snake and Tetris-like block-falling games are shown in Fig. 4b. Holograms calculated from *V*_logic_ (Fig. 4c) show good agreement with the experimentally reconstructed holograms (Fig. 4d).

We first demonstrate gameplay using the classic snake game (Supplementary Movie 2). The user controls the snake’s heading direction via the gamepad to chase a blinking apple pixel. When the snake consumes the apple, its length increases by one pixel, and a new apple is generated at a randomly selected unoccupied pixel. The game terminates when the snake undergoes self-collision, defined as an overlap between the snake’s head and its body. To quantify dynamic addressability, we record the spatiotemporal intensity evolution of all 36 pixels during a representative gameplay session (Fig. 4e). The resulting heatmap (pixels indexed *p* = 1–36 in Fig. 4a) indicates that each pixel is switched independently with minimal electrical crosstalk. Distinct dynamic events are clearly resolved: snake motion appears as diagonal or axial trajectories in the intensity map (e.g., transitions from pixel 36 to 30, 25, 19 or from 13 to 18 between 5 and 10 s), while snake-growth events manifest as abrupt increases in the number of bright pixels (Fig. 4e and corresponding sequences in Fig. 4f, rows 1–3). The apple pixel, periodically toggled at a 300 ms interval, appears as isolated recurrent intensity spikes (e.g., pixels 1 and 2 in Fig. 4e). The final frame (Fig. 4e and row 4 in Fig. 4f) captures the game-over state triggered by self-collision. Pixel contrast ranges from 2.4:1 to 7.3:1 (Supplementary Fig. 14), with the observed variations mainly attributed to differences in proximity to the zero-order reflection beam.

We further demonstrate a Tetris-like block-falling game (Supplementary Movie 3). Five canonical block geometries (Z, T, L, O, and I) are rendered using distinct spatial arrangements of the pixels. A single block is generated at the top row and descends under simulated gravity at an 800 ms refresh interval. Users may rotate or laterally shift the active block before it locks upon contacting either the bottom boundary or previously settled blocks. When a row becomes fully occupied, it is cleared and all rows above shift downward by one position, creating space for subsequent blocks. The game terminates when a newly generated block can no longer enter the field. Representative holographic frames for Z- and T-shaped blocks, including lateral shifting, rotation, and the game-over configuration, are shown in Fig. 4g. Across multiple rounds and mode transitions, the metadevice exhibits stable switching behavior and consistent optical output (Supplementary Movies 2, 3 and Supplementary Fig. 14). An encapsulated metadevice can operate for over 1200 switching cycles with only a ~2% reduction in intensity contrast (Supplementary Fig. 15, Supplementary Note 4 and Supplementary Fig. 16). Owing to its independently addressable 6 × 6 architecture, the device can in principle realize 2^36^ distinct holographic states, offering a large addressable optical state space for dynamic content generation beyond static metasurface implementations.

## Discussion

In this work, we have demonstrated interactively addressable organic metadevices that integrate electrochemical modulation of nanoscale antennas with electronically programmable, user-driven holographic functionality. This capability is distinct from prior electrochromic plasmonic systems, which primarily focused on plasmonic color or brightness modulation, or active-matrix display addressability without dynamic wavefront engineering (Supplementary Table 2)^63,64^. The platform functions as a class of intrinsically active projection displays, in which each pixel is independently switched through direct electrical addressing. The experimental demonstrations illustrate how electronically isolated metasurface pixels can be coordinated through software-defined voltage patterns, enabling functionalities that span from character rendering to interactive gameplay.

The presented platform offers several directions for further development. Increasing the pixel density through optimized nanoantenna design and higher-resolution electrode routing could enable more complex and high-fidelity holographic content^5,6^. Scaling the 2D in-plane fan-out arrangement toward higher-density arrays will require further optimization of the routing scheme and fabrication throughput. Improvements in redox kinetics and polymer engineering may further enhance the switching speed and operational bandwidth^65,66^. Future implementation using gel or solid-state electrolytes may improve device portability and environmental robustness, with further optimization of ion transport and switching speed^36,67–69^. More broadly, this work positions organic metasurfaces as a promising route toward low-voltage, high-density, and user-interactive photonic systems.

## Methods

### Numerical simulation and holography design

Finite-element simulations of the anomalous reflection intensity from the organic metasurfaces were carried out using COMSOL Multiphysics (see Supplementary Note 2 for details). For computer-generated holography, target images used for the character display and the interactive game display were discretized into 16 and 36 spatial segments, respectively, with each segment assigned to a single metasurface pixel. For each segment, a phase-only hologram was calculated using the Gerchberg–Saxton algorithm with 200 iterations^70^. The phase profiles were encoded into the AuNR orientations using an eight-level PB phase scheme^57–59^. For representative simulated frames (Figs. 2g and 4c), the phase profiles of metasurface pixels in their on state were combined to obtain the simulated far-field intensity distributions, corresponding to the holographic output of the metadevice.

### Metadevice fabrication

The ITO fan-out electrodes were fabricated on a Si/SiO_2_ (100 nm)/ITO (30 nm) substrate by direct laser writing (DMO-MicroWriter-3) with a positive photoresist (AZ 5214-E), followed by development in AZ 726 MIF. The patterns were transferred into the ITO layer by reactive ion etching (PlasmaPro 80, Oxford Instruments) using Ar and CF_4_ gases, and the residual resist was removed with acetone. AuNRs were fabricated by electron-beam lithography (EBL; Raith eLINE Plus) using a double-layer poly(methyl methacrylate) (PMMA) resist (AR-P 642.06, 200 K and AR-P 672.02, 950 K; Allresist). A 3 nm Cr adhesion layer and a 50 nm Au layer were deposited by electron-beam evaporation (Pfeiffer Classic 500 L), followed by lift-off in N-ethyl-2-pyrrolidone (NEP; AR 300-72, Allresist). PMMA masks for subsequent electrochemical polymerization were defined in an additional EBL step using computer-assisted alignment.

Electrochemical polymerization of PANI was carried out in a custom-built electrochemical cell containing an electrolyte solution of 2 M HNO₃ and 0.1 M aniline. A silver/silver chloride (Ag/AgCl) reference electrode and a platinum (Pt) wire counter electrode were used. During polymerization, all metasurface pixels were collectively cycled between −0.2 V and +0.8 V (vs. Ag/AgCl) at a scan rate of 25 mV s^−1^ using an electrochemical workstation (SP-200, Biologic). Polymerization was terminated after 65 cycles.

### Implementation of the electronic control loop

For device operation, the metadevice was placed in the electrochemical cell filled with 0.5 M HNO_3_ electrolyte. The ITO fan-out electrodes were connected to a custom-built sample holder (IMS CHIPS, Stuttgart) to provide independent electrical access to each metasurface pixel. A Pt wire counter electrode was held at ground. Pixel voltages were supplied by a McDAC controlled by a laptop running custom Python-based GUI software (Figs. 3b and 4b). User input from peripherals (keyboard or gamepad) was monitored by the control software and translated into voltage vectors that were applied across the pixel array to update the holographic output.

### Optical measurements

Metadevices were illuminated at normal incidence using a collimated 633 nm laser diode (Thorlabs, HL63163DG). CPL was generated using an LP and a QWP. The light beam was expanded using a pair of lenses to uniformly illuminate the metasurface array. Reconstructed holograms were projected onto a distant screen and recorded using a visible-light camera. Switching dynamics (Fig. 3c) were characterized by monitoring the intensity of the reconstructed hologram using a power meter (Thorlabs, S130C) placed at the screen plane during voltage modulation between −0.2 V (on, reduced state) and +0.6 V (off, oxidized state). Spatiotemporal intensity evolution was extracted from videos acquired at 24 frames per second by tracking the mean intensity within the hologram region corresponding to each pixel over time (regions marked by red squares and indexed consistently across the analysis). Intensities were normalized to the maximum on-state intensity across the array. The intensity contrast was defined as *I*_reduced_/*I*_oxidized_, where *I*_reduced_ and *I*_oxidized_ are the pixel intensities in the reduced (on) and oxidized (off) states, respectively. Pixel-resolved visibility metrics for the 6 × 6 projected pixel matrix were obtained from images acquired with all pixels in the on state (see Supplementary Note 3).

### Metadevice packaging

For an integrated prototype demonstration and quantitative evaluation of device performance, the metadevice was encapsulated using an ITO/SiO_2_ substrate adhered to the metasurface substrate with a UV-curable adhesive (Norland NOA 81) containing 7-μm glass spacer beads. After UV curing, the device was infiltrated with 0.5 M HNO_3_ electrolyte. The ITO layer, in contact with the electrolyte, served as the counter electrode and was connected to the grounded channel of the McDAC. The ITO fan-out electrodes were interfaced with a flat flexible ribbon cable (AWM 20624) through a custom-built sample holder, enabling independent electrical addressing of each metasurface pixel.

## Supplementary information

Supplementary information

Description Of Additional Supplementary File

Supplementary Movie 1

Supplementary Movie 2

Supplementary Movie 3

Transparent Peer Review file

## Source data

Source data

## Data Availability

All the data supporting the results of this study are included within the paper and the Supplementary Information. Source data are provided with this paper.

## Code availability

The codes that support the results of this study are available from the corresponding author upon request.
