# Supplementary material for: Interactively addressable organic metadevices
Source: Nat Commun. 2026 Jul 16;17:6251. doi: 10.1038/s41467-026-75757-4 (PMC13376165; doi:10.1038/s41467-026-75757-4)
Supplement: Supplementary file 1 — Supplementary information [file 41467_2026_75757_MOESM1_ESM.pdf]

Interactively addressable organic metadevices

Xiangyu Huang[1,2]*, Benjamin Renz*[1,2]*, Yueqiang Hu*[3]*, Na Liu*[1,2*]

[1] 2nd Physics Institute, University of Stuttgart, Pfaffenwaldring 57, 70569 Stuttgart, Germany
[2] Max Planck Institute for Solid State Research, Heisenbergstrasse 1, 70569 Stuttgart, Germany
[3] College of Mechanical and Vehicle Engineering, Hunan University, Changsha, P.R. China

Email: na.liu@pi2.uni-stuttgart.de

Supplementary Note 1: Fabrication process of interactively addressable organic metadevices

A schematic illustration of the step-by-step fabrication process of the organic metadevices is shown in Supplementary Fig. 1a. Indium tin oxide (ITO) fan-out electrodes and coarse alignment markers were fabricated by direct laser writing followed by reactive ion etching (RIE). Gold nanorod (AuNR) arrays and fine alignment markers were subsequently defined by electron-beam lithography (EBL) using poly(methyl methacrylate) (PMMA) resist, followed by Au deposition and lift-off (i–ii). To suppress pixel crosstalk during electrochemical polymerization, PMMA partition masks were defined in an additional EBL step using the fine alignment markers (iii). The PMMA masks spatially confine electrolyte access and thereby enable localized PANI deposition on the AuNR metasurface pixels (iv). After electrochemical polymerization, the PMMA masks were removed prior to device packaging.

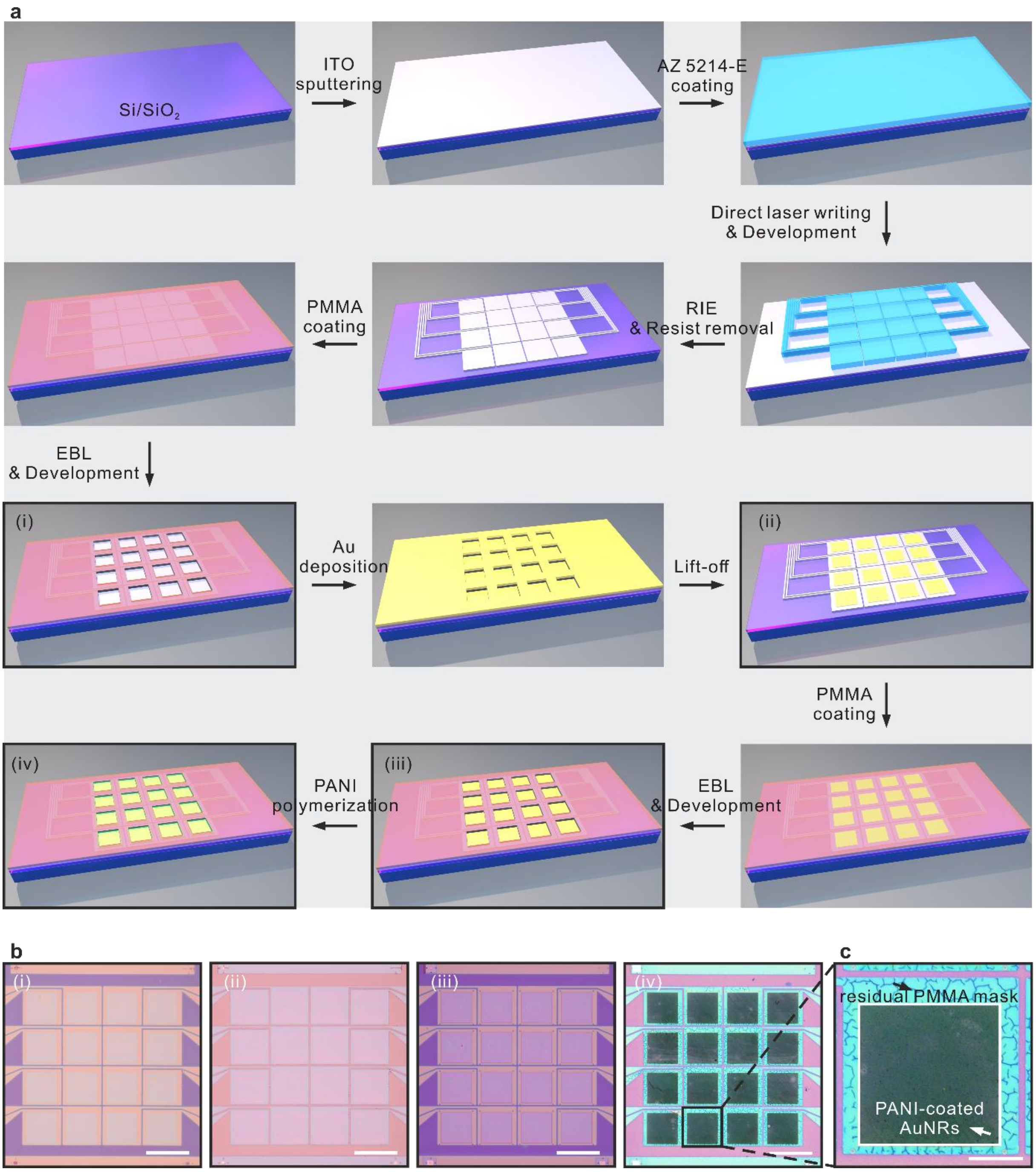

Supplementary Fig. 1 | Fabrication process of interactively addressable organic metadevices. a, Schematic illustration of the step-by-step fabrication process. **b**, Optical microscopy images of the metasurface array at fabrication steps (i)–(iv) shown in (**a**). Scale bar, 350 μm. **c**, Magnified optical microscopy image of one metasurface pixel. Scale bar, 100 μm.

Supplementary Note 2: Numerical simulations of organic metasurfaces

Finite-element simulations of the anomalous reflection intensity from the organic metasurfaces were carried out using COMSOL Multiphysics. Periodic boundary conditions, waveguide ports, and perfectly matched layers were applied. The AuNR was placed on a Si/SiO_2 (100 nm)/ITO (30 nm) substrate with a periodicity of 300 nm in both the *x*- and *y*- directions. Normally incident circularly polarized light (CPL) at 633 nm was defined with an electric field ($E_x = 1$, $E_y = i$, $E_z = 0$) and cross-polarized reflectance was recorded as the anomalous reflection intensity. The dielectric function of Au was taken from Johnson and Christy[1]. At 633 nm, the complex refractive indices were set to Si ($n = 3.88$, $k = 0.02$)[2], SiO_2 ($n = 1.5$, $k = 0$), ITO ($n = 1.78$, $k = 0.003$)[3], and the aqueous electrolyte ($n = 1.33$, $k = 0$). The refractive indices of PANI were set to $n = 1.63$, $k = 0$ in the reduced state and $n = 1.10$, $k = 0.27$ in the oxidized state, respectively[4,5].

The simulations support binary on/off modulation of anomalous reflection intensity between the reduced and oxidized states (Supplementary Fig. 2 a,b). The fabricated AuNR dimensions reported in the main text fall within the optimized parameter region identified by the simulations. Using the AuNR geometry (220 nm × 110 nm × 50 nm), we constructed a shell-like coating model to represent localized and conformal PANI growth around individual AuNRs, with a lateral shell thickness of 75 nm. We then simulated the anomalous reflection intensity and intensity contrast between the two redox states as a function of the total structural height *h*, which includes the 50 nm AuNR height and the vertical PANI thickness (Supplementary Fig. 2c). The intensity contrast was defined as $I_{reduced}/I_{oxidized}$, where $I_{reduced}$ and $I_{oxidized}$ denote the anomalous reflection intensities in the reduced (on) and oxidized (off) states, respectively. The simulated contrast increases with increasing *h*. However, excessively thick PANI coatings may introduce delamination and slower electrochemical switching. Balancing

optical performance and fabrication considerations, h = 120-165 nm was selected, corresponding to simulated contrast ratios of 2.2:1 to 3.3:1.

Simulated electric-field distributions of the PANI-coated AuNR (h = 165 nm) further support the conclusion that redox transitions of PANI modulate the plasmonic resonance and scattering behavior of the AuNRs. As shown in Supplementary Fig. 3, a large portion of the electric field extends outside the AuNR and dissipates within the surrounding dielectric environment, namely, the PANI shell. In the reduced state, the PANI-coated AuNRs exhibit pronounced plasmonic scattering, whereas in the oxidized state, increased absorption in the PANI shell suppresses the scattering intensity.

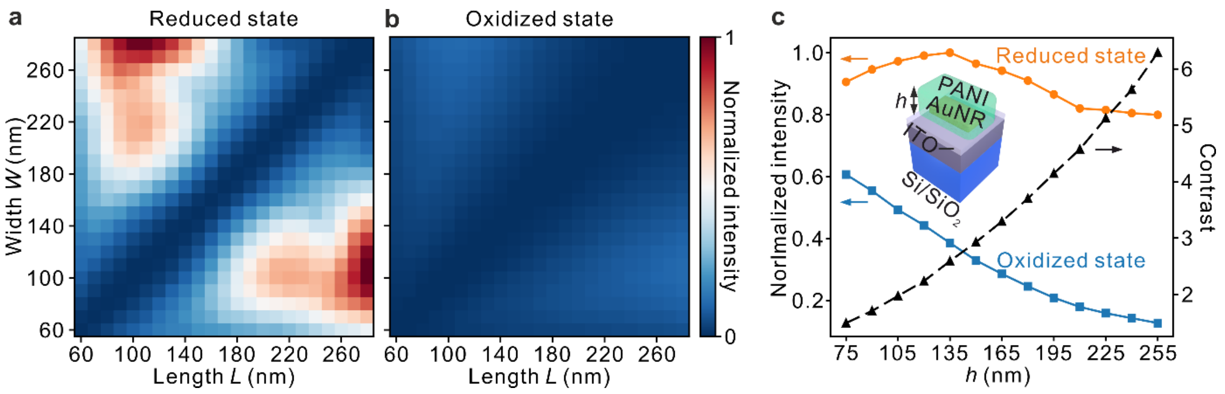

Supplementary Fig. 2 | Numerical simulations of organic metasurfaces. a,**b**, Simulated anomalous reflection intensity as a function of AuNR length L and width W (height fixed at 50 nm) for PANI-coated AuNRs with h = 165 nm in the reduced (**a**) and oxidized (**b**) states. Intensities are normalized to the maximum value. **c**, Simulated anomalous reflection intensity (left axis) and intensity contrast (right axis) as a function of h. The inset shows a schematic of the organic metasurface unit cell.

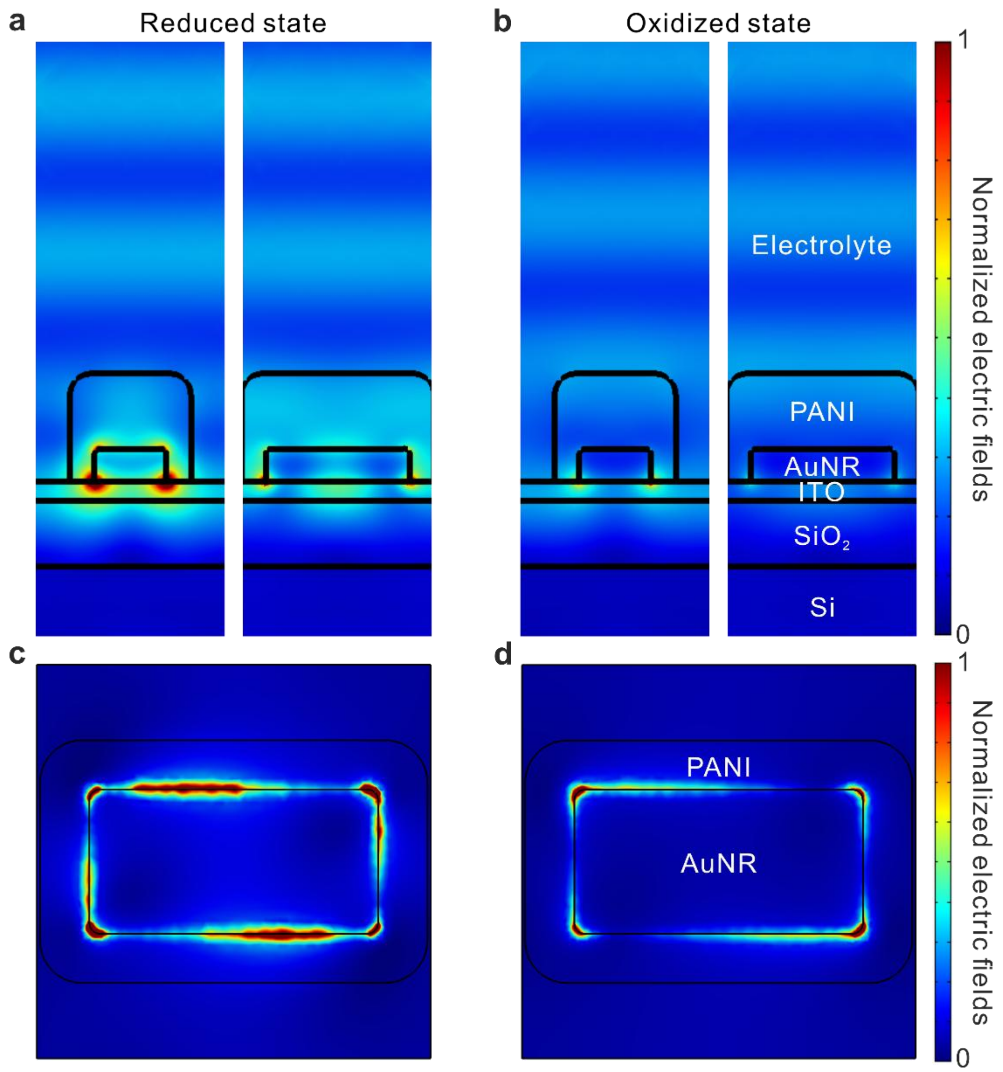

Supplementary Fig. 3 | Simulated electric-field distributions of organic metasurfaces. a,**b**, Electric-field distributions in two orthogonal cross-sectional planes through the center of the AuNR for the reduced (**a**) and oxidized (**b**) states. **c**,**d**, In-plane electric-field distributions of the AuNR for the reduced (**c**) and oxidized (**d**) states. The AuNR (220 nm × 110 nm × 50 nm) was coated with PANI with a lateral shell thickness of 75 nm and h = 165 nm.

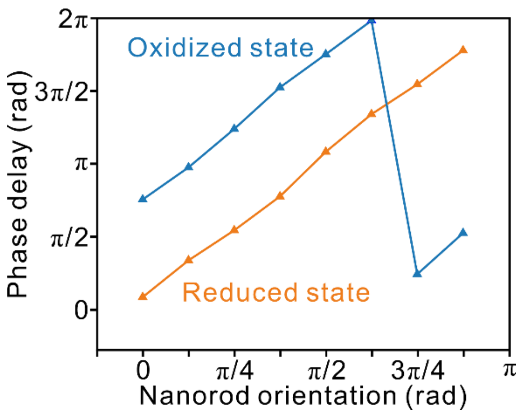

Supplementary Fig. 4 | Simulated phase profiles for reduced and oxidized PANI states. Simulated phase profiles as a function of AuNR orientation for reduced and oxidized PANI states (h = 165 nm).

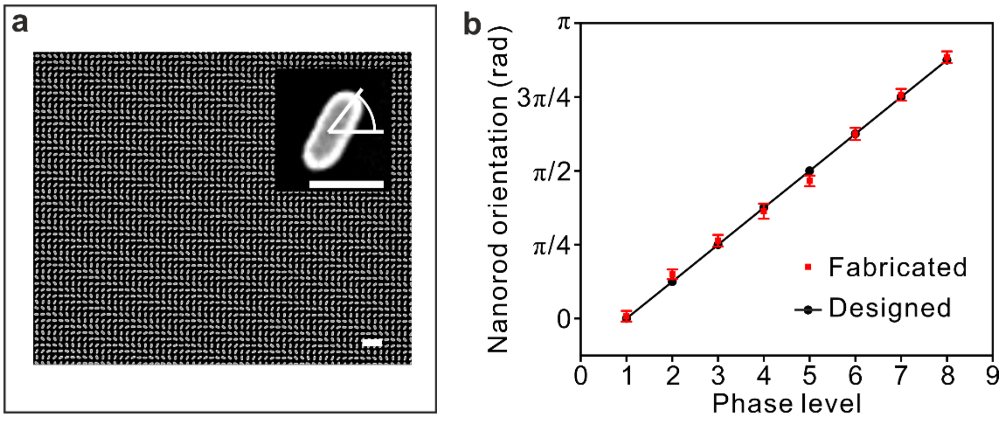

Supplementary Fig. 5 | Characterization of AuNR orientations. a, SEM image of the metasurface used for the statistical analysis of AuNR orientations. Scale bar, 1 μm. Inset: magnified SEM image of a single AuNR. Scale bar, 200 nm. **b**, Orientation uniformity analysis. The discrete orientations of AuNRs corresponding to eight PB phase levels were statistically extracted and compared with the designed values, showing good agreement with the intended phase encoding. Error bars denote the standard deviation of the AuNR orientations assigned to each PB phase level, as extracted from the SEM image.

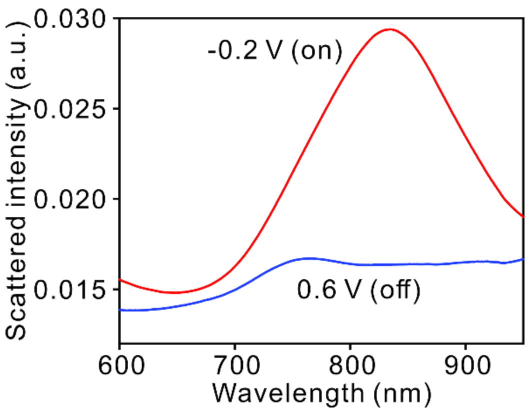

Supplementary Fig. 6 | Measured scattering spectra of a PANI-coated AuNR. In the reduced state at -0.2 V, the AuNR exhibits a plasmonic resonance (red curve), whereas in the oxidized state at +0.6 V, the resonance is strongly suppressed (blue curve).

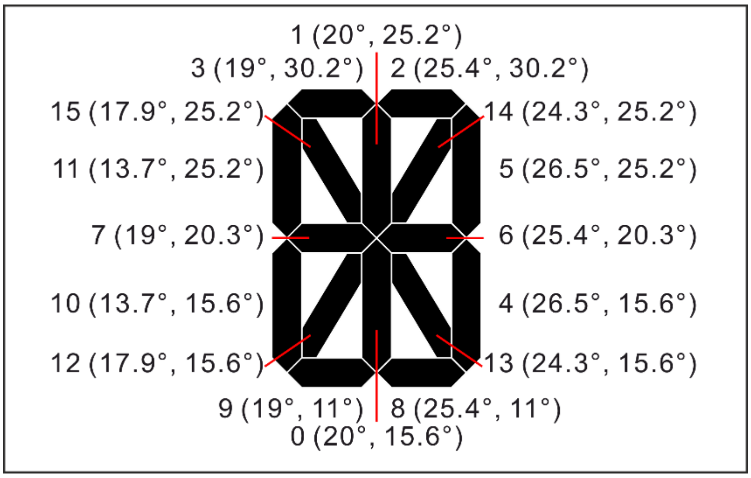

Supplementary Fig. 7 | Angular distribution information. Segments in holographic alphanumeric symbol generated by the 4 × 4 array of metasurface pixels.

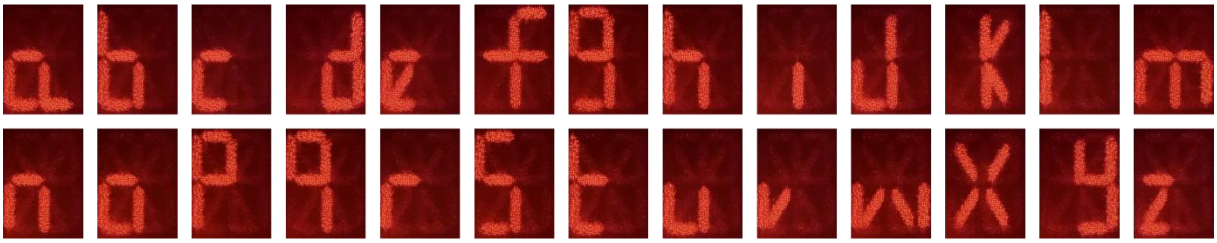

Supplementary Fig. 8 | Experimentally reconstructed holograms of lowercase letters.

Supplementary Table 1 | Look-up table of 62 alphanumeric characters

Character	Metasurface pixels in on state	Character	Metasurface pixels in on state
A	2, 3, 4, 5, 6, 7, 10, 11	f	0, 1, 2, 6, 7
B	0, 1, 2, 3, 4, 5, 6, 8, 9	g	0, 1, 3, 7, 9, 11
C	2, 3, 8, 9, 10, 11	h	0, 7, 10, 11
D	0, 1, 2, 3, 4, 5, 8, 9	i	0
E	2, 3, 10, 11, 7, 8, 9	j	0, 1, 9, 10
F	2, 3, 7, 10, 11	k	0, 1, 13, 14
G	2, 3, 4, 6, 8, 9, 10, 11	l	10, 11
H	4, 5, 6, 7, 10, 11	m	0, 4, 6, 7, 10
I	0, 1, 2, 3, 8, 9	n	0, 7, 10
J	4, 5, 8, 9, 10	o	0, 7, 9, 10
K	7, 10, 11, 13, 14	p	1, 3, 7, 10, 11
L	8, 9, 10, 11	q	0, 1, 3, 7, 11
M	4, 5, 10, 11, 14, 15	r	7, 10
N	4, 5, 10, 11, 13, 15	s	0, 3, 7, 9, 11
O	2, 3, 4, 5, 8, 9, 10, 11	t	7, 9, 10, 11
P	2, 3, 5, 6, 7, 10, 11	u	0, 9, 10
Q	2, 3, 4, 5, 8, 9, 10, 11, 13	v	10, 12
R	2, 3, 5, 6, 7, 10, 11, 13	w	4, 10, 12, 13
S	2, 3, 4, 6, 7, 8, 9, 11	x	12, 13, 14, 15
T	0, 1, 2, 3	y	1, 4, 5, 6, 8
U	4, 5, 8, 9, 10, 11	z	7, 9, 12
V	10, 11, 12, 14	0	2, 3, 4, 5, 8, 9, 10, 11, 12, 14
W	4, 5, 10, 11, 12, 13	1	4, 5, 14
X	12, 13, 14, 15	2	2, 3, 5, 6, 7, 10, 8, 9
Y	0, 14, 15	3	2, 3, 4, 5, 6, 8, 9
Z	2, 3, 8, 9, 12, 14	4	4, 5, 6, 7, 11
a	0, 7, 8, 9, 10	5	2, 3, 7, 8, 9, 11, 13
b	0, 7, 9, 10, 11	6	2, 3, 4, 6, 7, 8, 9, 10, 11
c	7, 9, 10	7	2, 3, 4, 5
d	0, 4, 5, 6, 8	8	2, 3, 4, 5, 6, 7, 8, 9, 10, 11
e	7, 9, 10, 12	9	2, 3, 4, 5, 6, 7, 8, 9, 11

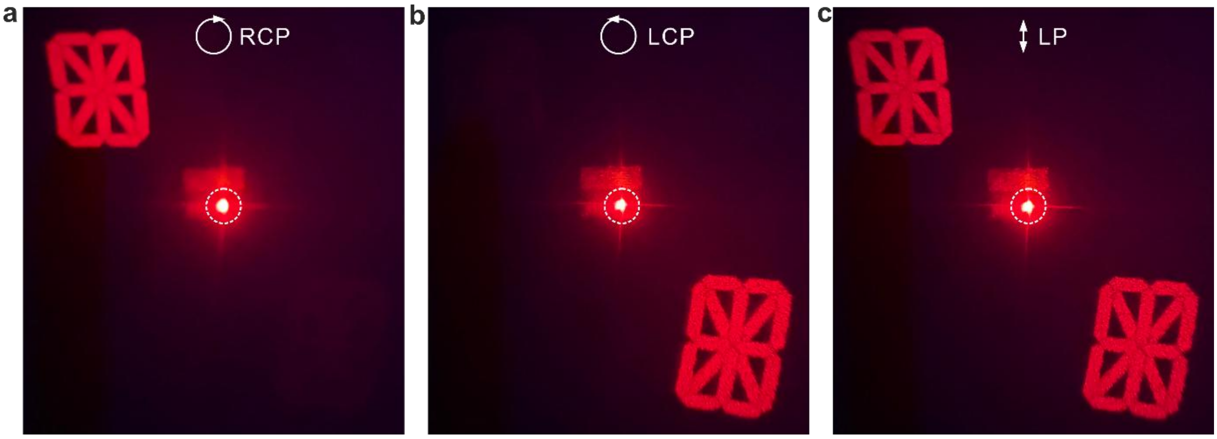

Supplementary Fig. 9 | Polarization dependence of the hologram. Experimental holographic projections under RCP, LCP, and LP illumination. Under LP illumination, both helicity channels are simultaneously excited, resulting in superposed holographic projections.

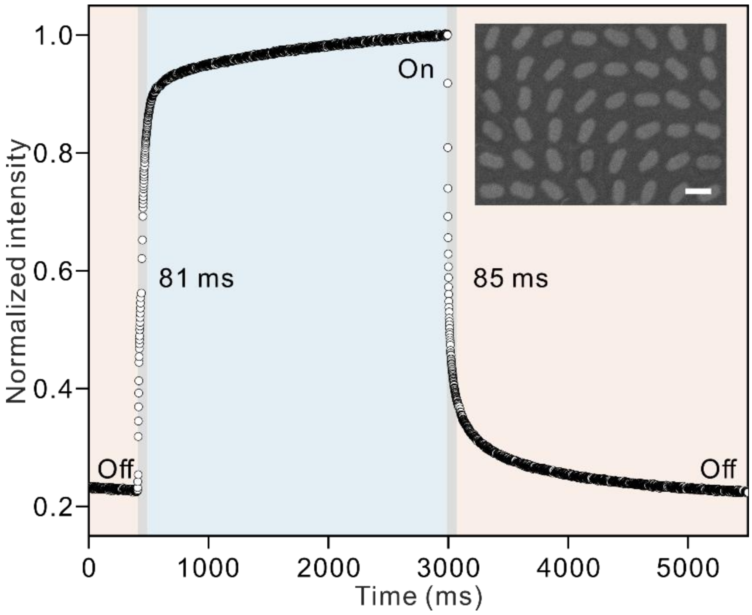

Supplementary Fig. 10 | Switching performance of the metadevice with increased PANI thickness. Temporal response of a metasurface pixel with a thicker PANI coating (250 nm) under alternating voltage modulation between -0.2 V (on) and +0.6 V (off). The thicker PANI coating improves the intensity contrast to 5.2:1, while increasing the switching times to 81 ms and 85 ms for the off-to-on and on-to-off transitions, respectively.

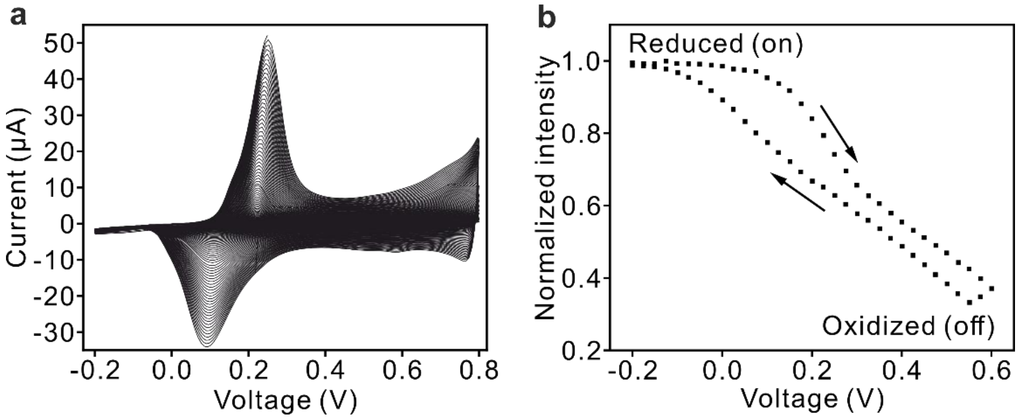

Supplementary Fig. 11 | Electrochemical characterization of the metadevice. a, Cyclic voltammetry response of the PANI-coated metasurface. **b**, Voltage-dependent anomalously reflected intensity, showing reversible switching between the reduced (on) and oxidized (off) states of PANI.

Supplementary Note 3: Evaluation of the holographically projected pixel array

Holographic projections from the organic metadevices were formed on a screen positioned 100 mm from the metadevice and recorded using a visible-light camera. The projected 36 holographic pixels span approximately 50 mm × 50 mm on the screen (Supplementary Fig. 12a,b). The outermost holographic pixels correspond to off-axis projection angles of approximately ±34° and ±33° along the horizontal and vertical directions, respectively, corresponding to an estimated field of view (FOV) of ~68° × 66°.

The metadevice exhibits a diffraction efficiency of 1.2%, defined as the intensity ratio between the anomalously reflected light and the incident circularly polarized light. The polarization conversion efficiency is 6%, defined as the intensity ratio between the cross-polarized reflected light and the total reflected light under circularly polarized illumination.

To quantitatively evaluate pixel-to-pixel visibility and optical uniformity, we computed pixel-resolved contrast-to-noise ratio (CNR) and signal-to-noise ratio (SNR) from the acquired RAW images.

The CNR and SNR were defined as[6]

$$\mathrm{CNR} = \frac{\langle I_\mathrm{S} \rangle - \langle I_\mathrm{B} \rangle}{\sigma_\mathrm{S}} \qquad (1)$$

$$\mathrm{SNR} = \frac{\langle I_\mathrm{S} \rangle}{\sigma_\mathrm{S}} \qquad (2)$$

where $\langle I_\mathrm{S} \rangle$ is the mean intensity within the signal region of interest (ROI, red square in Supplementary Fig. 12b) of a projected pixel, and σ_S is the standard deviation of the intensity within the same ROI. $\langle I_\mathrm{B} \rangle$ denotes the mean intensity of the dark background region. CNR describes the detectability of a bright pixel relative to the background, while SNR quantifies the robustness of the pixel signal against intensity variations (e.g., speckle) within the signal ROI.

For each pixel in the matrix, CNR and SNR were computed for each recorded frame and averaged over five frames. The resulting spatial maps show CNR values ranging from 2.03 to 2.49, and SNR values ranging from 2.07 to 2.55 (Supplementary Fig. 12c,d). These results indicate comparable pixel visibility across the array[6], supporting spatially uniform projection quality during interactive gameplay.

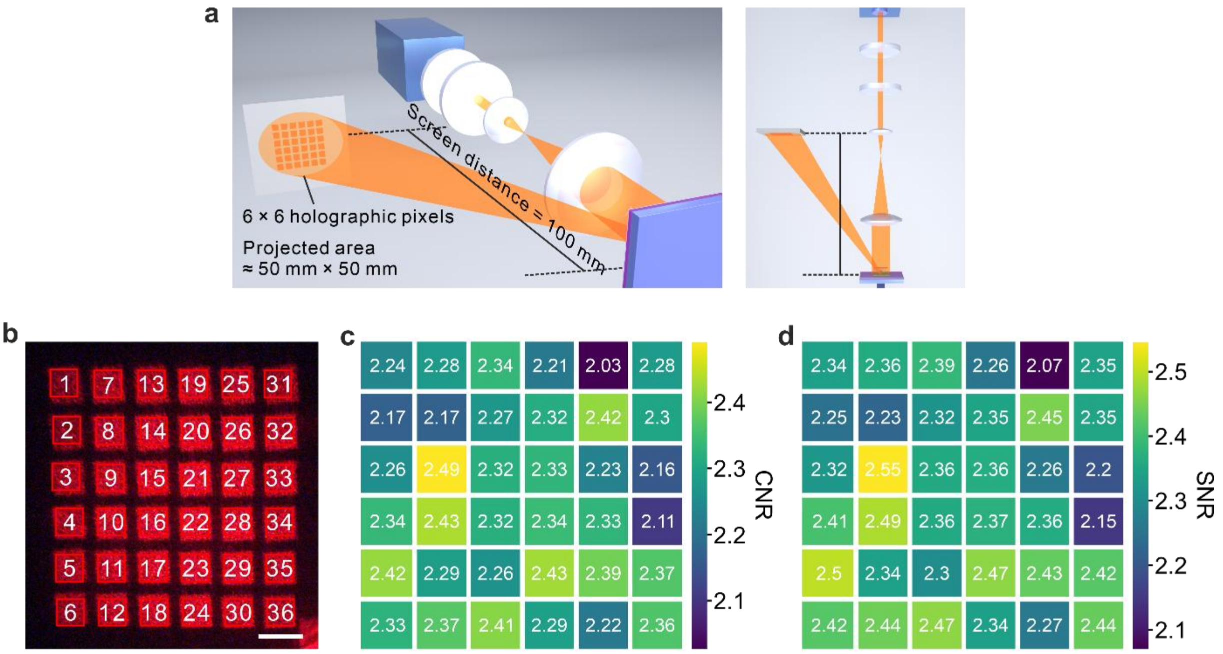

Supplementary Fig. 12 | Evaluation of the holographically projected pixel array. a, Schematic illustration of the optical measurement setup for the 6 × 6 pixel array. **b**, Representative holographic projection of the 6 × 6 pixel array on the screen. The hologram region of each pixel is marked by a red square with the corresponding pixel index used for analysis. Scale bar, 10 mm. **c**, Spatial maps of pixel-wise CNR and **d**, SNR across the 6 × 6 metasurface pixel array, extracted from the hologram regions and the dark background in (**b**) and averaged over 5 holographic projection images.

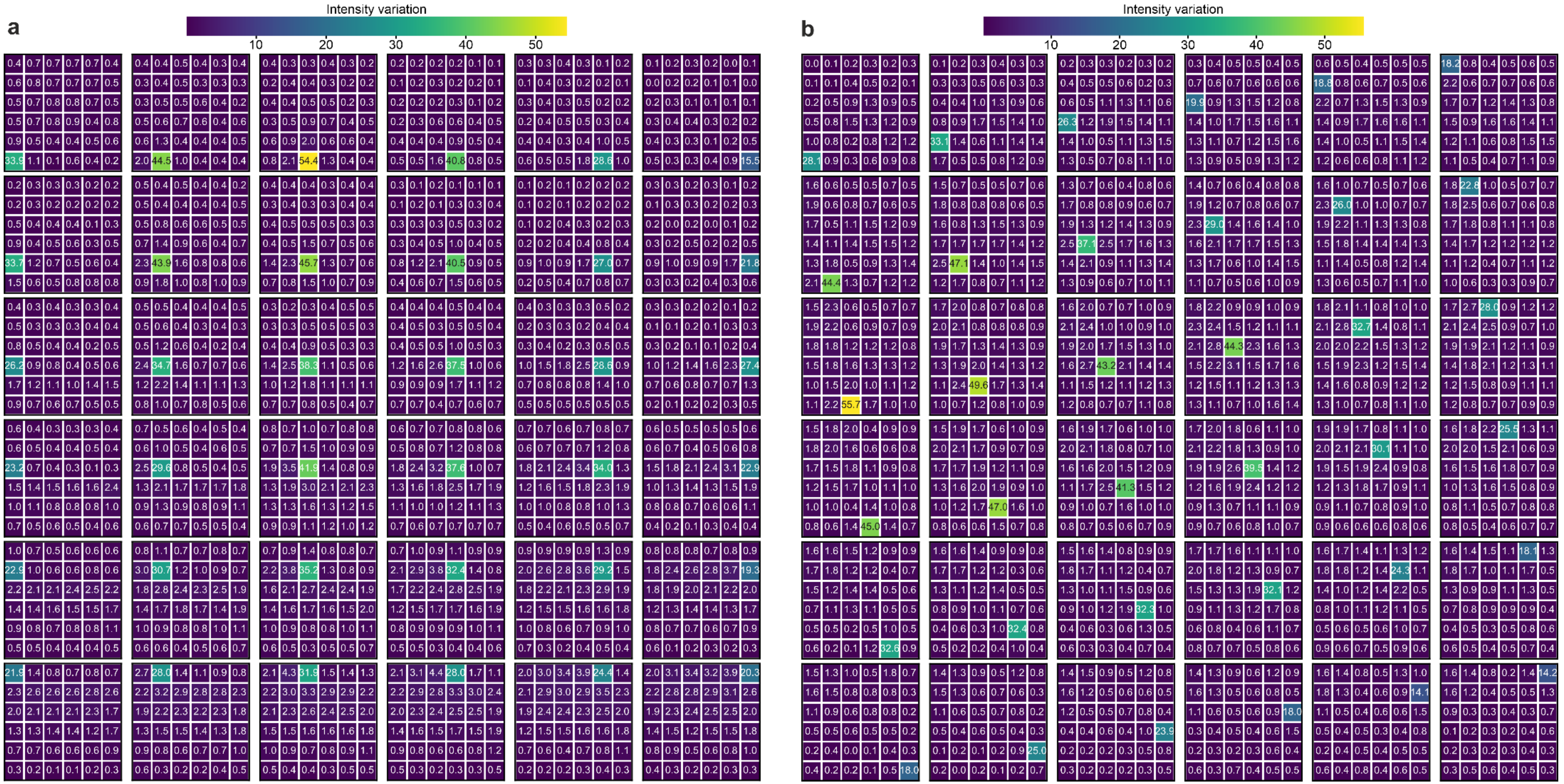

Supplementary Fig. 13 | Crosstalk evaluation of the pixel array. Intensity variations of all pixels during sequential switching of individual target pixels under row-by-row (**a**) and column-by-column (**b**) addressing sequences.

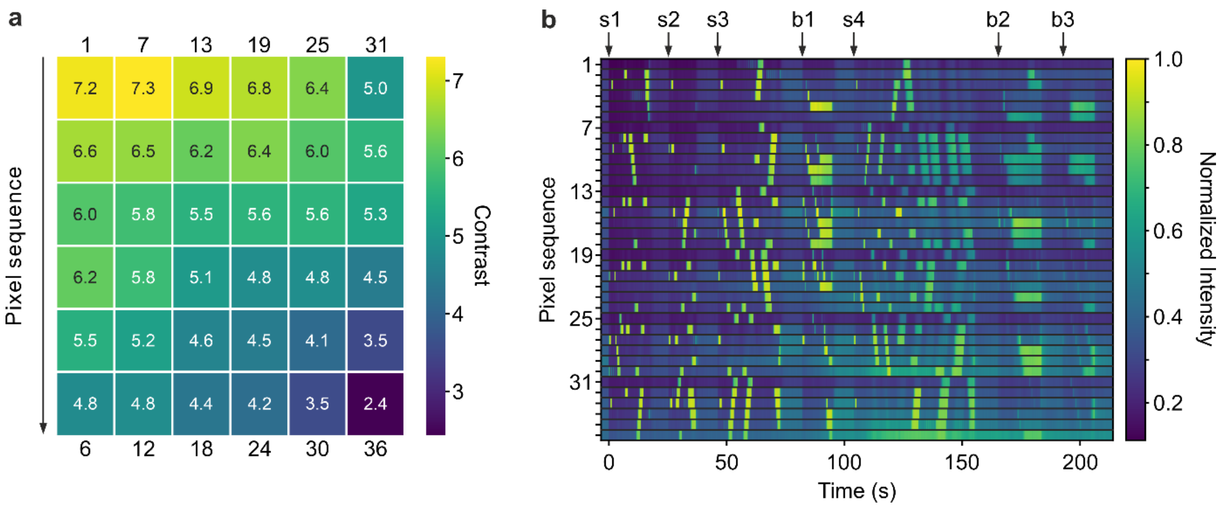

Supplementary Fig. 14 | Performance of the interactive holographic game platform. a, Spatial map of pixel-wise contrast across the 6 × 6 metasurface pixel array, extracted during interactive gameplay. Contrast values range from 2.4 to 7.3. The observed spatial variation primarily arises from the differences in the proximity of individual pixels to the zero-order reflection beam, which is located near the bottom-right corner. **b**, Spatiotemporal evolution of the normalized intensity for all 36 pixels during continuous interactive operation, including multiple game rounds and mode transitions. Each horizontal trace corresponds to a single metasurface pixel, and color scale encodes the normalized intensity. Arrows mark the start of individual game rounds with labels indicating snake game as s1-s4 and block-falling game as b1-b3, respectively.

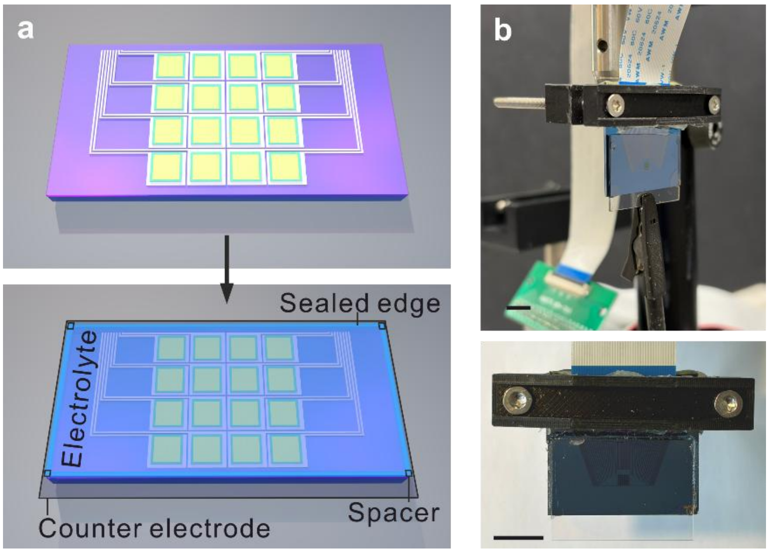

Supplementary Fig. 15 | Encapsulation of the organic metadevice. a, Schematic illustration of the metadevice encapsulation strategy. **b**, Photograph of the metadevice integrated with an ITO-glass counter electrode and electrolyte layer, mounted on a custom-built sample holder with external electrical interfacing. Scale bar, 1 cm.

Supplementary Note 4: Evaluation of operational stability

The encapsulated metadevice maintains stable electrochemical switching over 1200 switching cycles, with only a ~2% reduction in intensity contrast (Supplementary Fig. 16), demonstrating that the switching functionality is largely preserved during repeated operation. Minor intensity drift observed over extended cycling may originate from gradual electrochemical or structural changes in the conducting polymer layer. Previous studies have demonstrated substantially longer switching lifetimes in conducting polymer systems without significant degradation[7–9], suggesting that the operational durability of the organic metadevice could be further improved through materials optimization and device engineering.

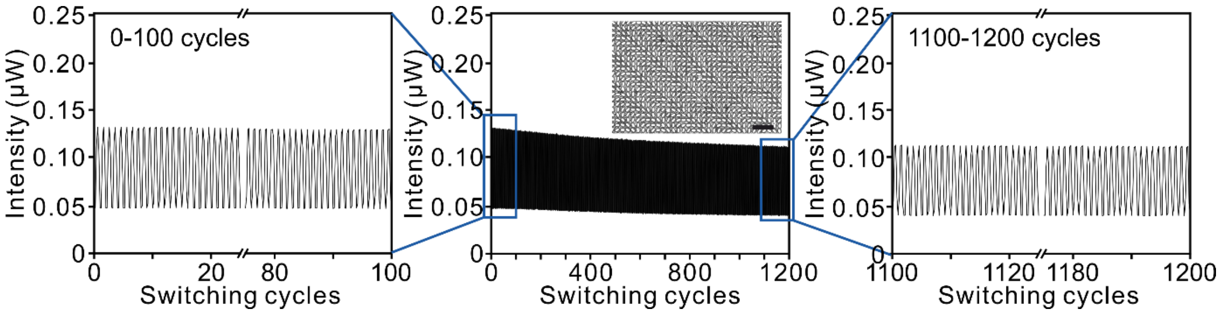

Supplementary Fig. 16 | Evaluation of operational stability. Anomalously reflected light intensity recorded from the metadevice while all pixels were simultaneously switched between -0.2 V (on) and +0.6 V (off), with each voltage applied for 2.5 s over 1200 switching cycles. The enlarged traces show representative switching cycles at the initial and final stages of the cycling test. The inset shows an SEM image of the PANI-coated AuNRs after cycling, revealing no obvious morphological degradation of the metasurface. Scale bar, 1 μm.

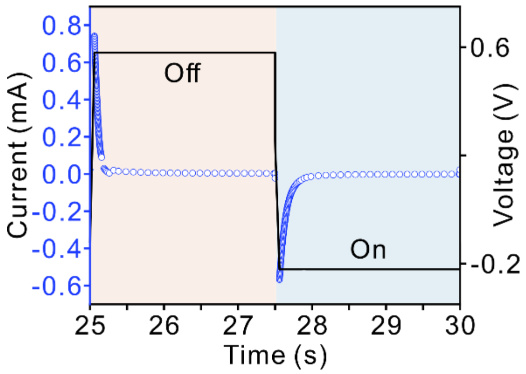

Supplementary Fig. 17 | Chronoamperometric characterization of electrochemical switching. Current response of the metadevice under alternating voltage modulation between +0.6 V (off) and -0.2 V (on), with each voltage applied for 2.5 s. The integrated current response was used to quantify the corresponding energy and power consumption during electrochemical switching. The corresponding energy and power consumption during electrochemical switching were quantified to be 1.58 mJ cm^{-2} and 0.630 μW cm^{-2} for oxidation (off), and 0.64 mJ cm^{-2} and 0.258 μW cm^{-2} for reduction (on), respectively.

Supplementary Table 2 | Comparison of the present work with representative electrochromic plasmonic systems

Aspect	This work	Ref. 10 (Sci. Adv. 2019, 5, eaaw2205)	Ref. 11 (RSC Appl. Interfaces 2024, 1, 719–727)
**System type**	Interactively addressable organic metadevice	Electrochromic plasmonic nanopixels	TFT-addressed electrochromic display
**Nanophotonic structure**	PB-phase metasurface based on PANI-coated AuNRs	PANI-coated Au nanoparticles	Fabry–Pérot electrochromic cavities
**Structural organization**	Lithographically defined metasurface pixels	Self-assembled plasmonic nanoparticle assemblies	TFT-integrated electrochromic structures
**Demonstrated architecture**	Independently addressable metasurface array	Uniformly modulated electrochromic plasmonic surface	TFT-addressed electrochromic display
**Optical functionality**	Dynamic holographic projection	Plasmonic coloration	Reflective color display
**Holographic wavefront engineering**	Yes	No	No
**Phase modulation**	Yes	No	No
**Interactive optical operation**	Yes	No	No
**Demonstrated functionality**	Interactive holographic projection and user-interactive operation	Plasmonic color switching	Active-matrix reflective color display
**Primary research focus**	Interactively programmable visible-wavelength metadevices	Electrochromic plasmonic coloration	Electrochromic display engineering

References

(1) Johnson, P. B. & Christy, R. W. Optical constants of the noble metals. *Phys. Rev. B* **6**, 4370–4379 (1972).

(2) Aspnes, D. E. & Studna, A. A. Dielectric functions and optical parameters of Si, Ge, GaP, GaAs, GaSb, InP, InAs, and InSb from 1.5 to 6.0 eV. *Phys. Rev. B* **27**, 985–1009 (1983).

(3) König, T. A. F. et al. Electrically tunable plasmonic behavior of nanocube–polymer nanomaterials induced by a redox-active electrochromic polymer. *ACS Nano* **8**, 6182–6192 (2014).

(4) Barbero, C. & Kötz, R. Nanoscale dimensional changes and optical properties of polyaniline measured by *in situ* spectroscopic ellipsometry. *J. Electrochem. Soc.* **141**, 859 (1994).
(5) Huang, X. et al. Organic metasurfaces with contrasting conducting polymers. *Nano Lett.* **25**, 890–897 (2025).
(6) Eliezer, Y. et al. Suppressing meta-holographic artifacts by laser coherence tuning. *Light Sci. Appl.* **10**, 104 (2021).
(7) Leroux, Y. R. et al. Conducting polymer electrochemical switching as an easy means for designing active plasmonic devices. *J. Am. Chem. Soc.* **127**, 16022–16023 (2005).
(8) Lu, W.; Menezes, L. d. S.; Tittl, A.; Ren, H. & Maier, S. A. Active Huygens' metasurface based on in-situ grown conductive polymer. *Nanophotonics* **13**, 39–49 (2024).
(9) Xiong, K. et al. Video speed switching of plasmonic structural colors with high contrast and superior lifetime. *Adv. Mater.* **33**, 2103217 (2021).
(10) Peng, J. et al. Scalable electrochromic nanopixels using plasmonics. *Sci. Adv.* **5**, eaaw2205 (2019).
(11) Olsson, O.; Gugole, M.; Blake, J. C.; Chukharkin, M. & Dahlin, A. Electrochromic active matrix with plasmonic metasurfaces. *RSC Appl. Interfaces* **1**, 719–727 (2024).
