# Supplementary material for: Interactively addressable organic metadevices
Source: Nat Commun. 2026 Jul 16;17:6251. doi: 10.1038/s41467-026-75757-4 (PMC13376165; doi:10.1038/s41467-026-75757-4)
Supplement: Supplementary file 2 — Description Of Additional Supplementary File [file 41467_2026_75757_MOESM2_ESM.pdf]

**Description of Additional supplementary files**

**Supplementary Movie 1**: Interactive holographic character display.

**Supplementary Movie 2**: Interactive holographic snake-game display.

**Supplementary Movie 3:** Interactive holographic block-falling game display.
