# Supplementary material for: Interactively addressable organic metadevices
Source: Nat Commun. 2026 Jul 16;17:6251. doi: 10.1038/s41467-026-75757-4 (PMC13376165; doi:10.1038/s41467-026-75757-4)
Supplement: Supplementary file 6 — Transparent Peer Review file [file 41467_2026_75757_MOESM6_ESM.pdf]

nature portfolio

Peer Review File

Interactively addressable organic metadevices

Corresponding Author: Professor Na Liu

This file contains all reviewer reports in order by version, followed by all author rebuttals in order by version.

Version 0:

Reviewer comments:

Reviewer #1

(Remarks to the Author)
The authors presented an interactively addressable organic metadevice platform based on the electrochemical redox modulation of conducting polymers. By conformally integrating plasmonic Au nanorod (NR) nanoantennas with a thin polyaniline (PANI) coating, the platform leveraged locally voltage-controlled redox-state changes that altered the complex refractive index and absorption of PANI, thereby enabling pixel-level binary ("on/off") modulation of the nanoantenna scattering amplitude. A planar indium tin oxide (ITO) fan-out architecture electrically isolated and independently drove each metasurface pixel in a two-dimensional array. Operating at visible wavelengths, the system demonstrated 4 x 4 and 6 x 6 independently addressable arrays, sub-volt actuation (-0.2 V / +0.6 V), and millisecond-scale switching (~24 ms and ~2 ms). Embedded in a closed-loop control scheme, user inputs from a keyboard or gamepad were translated in real time into pixel-resolved voltage vectors, enabling dynamic holographic character projection and interactive gaming, while confirming negligible electrical crosstalk, reasonably uniform pixel responses, and stable operation under evolving holographic scenes.

By elevating an electrochemically tunable metasurface from a material-level demonstration to a system-level, user-programmable interactive photonic platform, this work offered an intrinsic (non-external-SLM) route toward visible-wavelength, two-dimensional programmable wavefront control and holographic display. It further showcased software-defined voltage-pattern driving for reconfigurable content generation and a potentially large optical state space (e.g., 2^36 states for a 6 x 6 array). While pixel-level electrically addressed organic metasurfaces appeared promising for display/projection and reconfigurable photonic devices, the manuscript still benefited from stronger engineering metrics and broader validation. Overall, it constitutes a clearly novel platform-style proof of concept that can meet the high publication standards if key experiments and analyses are strengthened.

1. The device operation relies on a liquid electrolyte, which makes the work closer to a platform-level validation than an integrable product prototype. The authors could strengthen the manuscript by providing more data to support long-term operational stability and a clear packaging/encapsulation pathway.

2. The demonstrated arrays are limited to 4 x 4 and 6 x 6, with a 350 um pixel pitch, constituting a low-resolution proof of concept. Both the scale and image fidelity are therefore constrained and remain orders of magnitude away from high-fidelity holography/display, motivating additional evidence from higher-resolution arrays and pixel-level experiments.

3. Although the authors presented switching times and intensity contrast, the performance metrics are not yet comprehensive. In particular, a systematic, closed-loop evaluation of key engineering figures of merit, such as the diffraction efficiency, power consumption, cycling lifetime, and long-term drift, are still missing, with the current discussion relying more on the qualitative descriptions of "stable operation/low crosstalk."

4. The authors employed Pancharatnam-Berry (PB) phase metasurfaces to encode holographic phase profiles via orientation control of Au NRs. However, no characterizations of orientation uniformity, scattering spectra, phase gradient fidelity, or polarization conversion efficiency are provided. The device primarily operates via amplitude modulation through PANI absorption. Is phase modulation preserved during switching? How do the potential phase distortions affect the fidelity of holographic reconstruction?

5. The reasons of using circularly polarized light (CPL) should be clarified. The optical efficiency of the metadevices under CPL should be quantified. Can the devices operate under linearly polarized or unpolarized illumination?

6. Supplementary Fig. 1 presents the simulated on/off contrast ratio. However, the experimentally achieved on/off contrast is lower. Please explain the origin of this gap. Can the on/off contrast ratio be improved by optimizing the PANI thickness or oxidation state?

7. The electrochemical characteristics of the redox-driven switching of PANI such as cycling endurance and the correlation between voltage modulation and oxidation state are not provided. Basic electrochemical data should be included to support the switching mechanism.

8. The authors state that negligible electrical crosstalk is achieved, yet no supporting data are presented. Quantitative crosstalk characterization such as intensity leakage into neighboring off-pixels during switching should be included.

Reviewer #2

(Remarks to the Author)
This manuscript presents an interactively addressable 2D organic metadevice based on electrochemical modulation. The authors demonstrate a platform where ultrathin polyaniline (PANI) is conformally integrated with plasmonic gold nanorods (AuNRs) to create metasurface pixels that can be switched independently. By employing a planar fan-out ITO electrode architecture, the system achieves independent 2D pixel control without the crosstalk typical of liquid-crystal systems. The interdisciplinary nature of the work—combining micro-nanofabrication, polymer chemistry, and software-defined control—is well-suited for the broad readership of Nature Communications. Due to several issues, I recommend a revision of this manuscript.:
1. About the scalability and the Definition of "High-Density". The authors emphasize that this platform paves the way for "high-density" addressable 2D organic meta-optics. The current demonstration is limited to 4*4 and 6*6 arrays using a planar fan-out routing scheme. As the pixel count scales towards megapixels (or even 32*32), the real estate required for individual ITO leads in a single plane will inevitably lead to a drop in the optical fill factor or an unmanageable increase in chip footprint. Can the authors provide a quantitative analysis of the physical scaling limits of this fan-out architecture? Calling the current scheme a route to "high-density" requires a more rigorous boundary definition.
2. The system currently operates within an electrochemical cell filled with 0.5M~HNO3. While acceptable for a proof-of-concept, a "user-interactive" device operating in a strong acid environment faces severe challenges regarding packaging, component corrosion, and portability. Have the authors conducted any durability tests on the electrodes and AuNRs under prolonged exposure to this acidic environment? The authors mention solid-state electrolytes as a future direction ; however, if the ion diffusion rate drops significantly in a solid matrix, would the "millisecond-scale" switching still be achievable for real-time interaction?
3. The reported intensity contrast is approximately 3.5:1. What is the fundamental physical bottleneck for this relatively low contrast? Is it residual scattering in the oxidized PANI state or insufficient extinction of the plasmonic resonance? In the 6*6 array, did the authors observe any optical crosstalk? Specifically, when adjacent pixels are in different "On/Off" states, does the diffractive overlap affect the fidelity of the reconstructed holographic image?
4. About Electrochemical Stability and Cycle Life. Redox-active polymers often undergo volume changes during cycling, which can lead to delamination from metallic nanostructures. Did the authors perform long-term cycling tests (e.g., >1,000 or 10,000 cycles)? Please provide data or SEM imagery of the PANI-coated AuNRs after extensive switching to confirm the structural and functional integrity of the conformal coating.

Some references should be included in the manuscript:
① Li J, Yu P, Zhang S, et al. A reusable metasurface template[J]. Nano letters, 2020, 20(9): 6845-6851.
② Fan, X., Xiong, W., Xu, K. et al. Spatial light modulator via optically addressed metasurface. Nat. Nanotechnol. (2026). https://doi.org/10.1038/s41565-026-02128-x
③ Li X, Chen QM, Zhang X, Zhao RZ, Xiao SM et al. Time-sequential color code division multiplexing holographic display with metasurface. Opto-Electron Adv 6, 220060 (2023). DOI: 10.29026/oea.2023.220060

Reviewer #3

(Remarks to the Author)
What are the noteworthy results?
This study demonstrates a significant breakthrough in active metasurfaces by implementing a planar fan-out architecture that enables independent, pixel-level addressing of PANI-integrated plasmonic nanoantennas in the visible regime. The device achieves millisecond-scale switching dynamics while maintaining negligible pixel-to-pixel crosstalk. The practical utility of this platform is further validated by a user-driven, interactive holographic system capable of real-time rendering and interactive gaming, highlighting its potential for programmable, responsive photonic applications.

Will the work be of significance to the field and related fields? How does it compare to the established literature? If the work is not original, please provide relevant references.
While the manuscript demonstrates an integrated platform for interactive holographic displays, the overall conceptual novelty and scientific contribution appear somewhat limited for a journal of Nature Communications' standing. The work feels more like an incremental engineering integration of established materials and electronic designs rather than a fundamental breakthrough. My concerns are detailed as follows:
1. Incremental Material Innovation
The use of PANI-coated Gold Nanorods (AuNRs) as the active medium is not a novel structural or material advancement. Similar electrochemical modulation mechanisms and material architectures have already been extensively explored for

electrochromic and plasmonic display applications (e.g., https://www.science.org/doi/10.1126/sciadv.aaw2205).The authors fail to clearly differentiate how their specific implementation provides a superior or fundamentally different optical modulation mechanism compared to these well-established benchmarks.
2. Limited Advancement in Electronic Engineering
The electronic control strategy, while functional, follows conventional engineering practices.
• Addressing Scheme: Using a 16-pixel array for alphanumeric rendering is a standard approach in display technology and does not represent a significant leap in pixel density or addressing logic for metasurfaces.
• Integration: The methodology of integrating electrochemically active soft materials onto electronic controllers for individual pixel modulation has been previously demonstrated in similar contexts (e.g., https://pubs.rsc.org/en/content/articlehtml/2024/lf/d3lf00266g). The "planar fan-out" architecture presented here appears to be a practical optimization rather than a transformative engineering principle.
3. Disparity Between Engineering Application and Scientific Depth
The primary strength of the paper lies in its system-level application, specifically the integration with external peripherals for interactive gaming and real-time displays. However, this is largely an engineering effort. In its current form, the manuscript lacks a rigorous exploration of the underlying physics—such as the complex electrochemical-optical coupling dynamics at the organic-inorganic interface or the limits of phase/amplitude control in such hybrid systems. For a high-impact multidisciplinary journal, the scientific content feels "thin" compared to the technical implementation of the user interface.

Does the work support the conclusions and claims, or is additional evidence needed?
Generally, the experimental results align with the primary claims. However, the assertions regarding “negligible electrical crosstalk” and “stable operation” require more rigorous quantitative validation to be fully convincing.

Are there any flaws in the data analysis, interpretation and conclusions? Do these prohibit publication or require revision?
While the conceptual demonstration is interesting, several critical gaps in the experimental evidence and technical descriptions must be addressed. These issues, ranging from fabrication precision to system integration, currently limit the reproducibility and scientific rigor of the work.
1. Ambiguity in Pixel Fabrication and Isolation:
Although the Methods section mentions EBL and PMMA masking, the Main Text lacks a visual demonstration of this critical integration. Notably, in Figure 2d, there appear to be filamentous structures at the edges of the pixels; it is unclear whether these are metallic residues or PMMA resist remnants. To substantiate the claim of negligible crosstalk, the authors should provide a step-by-step fabrication schematic and images showing the precise confinement of PANI within the pixel boundaries. This is essential to confirm that no parasitic conductive paths are formed by overgrowth or fabrication artifacts.
2. Incomplete Electrochemical System Integration:
The electrochemical control mechanism is central to this work, yet its integration remains underspecified. While Figure 2c illustrates the interface between the control system and the display, the physical integration of the electrochemical cell (e.g., electrolyte containment, counter/reference electrode placement within the 2D array) is not clearly explained. This omission raises significant concerns regarding the practical feasibility and scalability of the "integrated" device.
3. Lack of Quantitative Holographic Parameters:
The description of the holographic projection system in Figure 3a is overly qualitative. Key parameters necessary for evaluating display performance are missing, such as:
• The precise distance between the metasurface and the screen.
• The physical dimensions of the projected image on the screen.
• The field of view (FOV).
Without these metrics, it is impossible to assess the practical utility or the resolution limits of the proposed display.
4. Insufficient Optical Characterization of AuNRs:
The optical characterization of the PANI-coated AuNRs is inadequate for a metasurface-focused study. Figure 1S alone does not provide sufficient data on the spectral response or the absolute intensity modulation during electrochemical cycling. The authors should also provide comprehensive extinction/scattering spectra. This is vital for evaluating the device’s performance as a dynamic display.
5. Photothermal Stability and Operational Lifetime:
Given the emphasis on "stable operation" and the use of a laser source, the impact of photothermal effects cannot be ignored. Gold nanorods are efficient light-to-heat converters; under continuous laser illumination, local heating may accelerate the degradation of the PANI coating or the electrolyte. The authors must provide lifetime testing data, specifically plotting contrast/intensity loss vs. cycle count. A stability test under actual operating conditions (laser-illuminated cycling) is necessary to support the claim of a "stable" interactive system.

Is the methodology sound? Does the work meet the expected standards in your field?
Not entirely. As noted in my previous comments, the methodology requires substantial upgrades to meet the field's standards. Specifically, the manuscript lacks quantitative assessments of pixel-level electrochemical crosstalk and rigorous stability testing under laser-induced photothermal conditions

Is there enough detail provided in the methods for the work to be reproduced?
No. The current "Methods" section lacks critical details necessary for reproduction, particularly regarding the precise localized deposition of AuNRs on pixel electrodes and the physical integration of the electrochemical cell within the 2D array. More transparent fabrication protocols and optical setup parameters are required.

Version 1:

Reviewer comments:

Reviewer #1

(Remarks to the Author)
The authors have thoroughly revised their manuscript and addressed the previous comments point by point. The revised version is of higher quality and easier to understand. The revised manuscript has provided substantially expanded data to demonstrate both the operational stability and encapsulation feasibility of the platform. The issues related to resolution and pixel level validation have been addressed through the additional proof of concept demonstrations, accompanied by clearer and more physically grounded interpretations. Moreover, the authors have systematically investigated the effect of the PANI thickness on the switching performance and have included electrochemical data to characterize the evolution of the PANI properties.

In addition, the revision has incorporated quantitative evaluations of the key engineering figures of merit, including the diffraction efficiency, power consumption, cycling lifetime, and long-term operational drift. To resolve concerns regarding the orientation uniformity and phase gradient fidelity, statistical analyses based on the SEM images of the metasurface have been introduced. Furthermore, the rationale for employing circularly polarized light has been clarified, and the corresponding optical efficiency has been experimentally quantified. A quantitative characterization of electrical crosstalk has also been added, supported by new measurements and detailed analysis.

Overall, the revision has resolved the substantive concerns and resulted in a coherent, technically sound article with clear takeaways for readers. I recommend acceptance in its current form.

Reviewer #2

(Remarks to the Author)
The authors have answered my questions.

Reviewer #3

(Remarks to the Author)
I thank the authors for their careful revisions and detailed responses to my comments. I am generally satisfied with the changes made in the revised manuscript.

First, I appreciate the authors' efforts to clarify the novelty and positioning of the work relative to the related studies I suggested. I agree with the statement that “Neither work focused on programmable metasurface optics, holographic wavefront reconstruction, or interactive metadevice operation in the visible regime.” The comparison with the prior literature helps to clearly establish the fundamental distinctions and contributions of the present study.

Second, regarding my concern about the limited theoretical analysis in the original submission, the authors have significantly strengthened the manuscript by providing detailed numerical investigations of the electrochemical–optical coupling mechanisms. The additional simulations and analyses relating to the optical scattering response to the geometry evolution offer valuable physical insight into the device operation.

Furthermore, I appreciate the authors' additional quantitative characterizations addressing my concerns regarding the claims of “negligible electrical crosstalk” and “stable operation.” The new experimental evidence adequately supports these statements and improves the overall rigor of the work.

Finally, the concerns I raised regarding the electrochemical setup have also been satisfactorily addressed through the newly added experimental images and corresponding discussions.

Overall, the authors have carefully addressed my major comments, and the revised manuscript has been substantially improved. I have no further major concerns. I only have two minor suggestions that may further improve the clarity and reproducibility of the work:

1. In Supplementary Fig. 1b(iv), I am still curious about the identity of the filament-like material observed between adjacent pixels. It would be helpful if the authors could clarify whether this feature corresponds to residual resist, PANI, or another material. Such information may provide additional insight into the protective role of PMMA during the electrochemical processes and could be of interest to readers.

2. For the holographic pixel setup, I would recommend adding a simple schematic illustration with clearly labeled dimensions and geometric parameters. While the current description is understandable, a dedicated schematic would allow readers to more intuitively visualize the device architecture and experimental configuration, thereby improving clarity and facilitating reproducibility.

Version 2:

Reviewer comments:

Reviewer #3

(Remarks to the Author)
I would like to thank the authors for their thorough and thoughtful revisions throughout the review process. I am very satisfied with the final version of the manuscript.

The authors have carefully addressed all of my concerns and have responded constructively to the suggestions raised during the review. The additional analyses, experiments, clarifications, and improvements to the presentation have significantly strengthened the manuscript and enhanced its clarity.

I appreciate the authors' efforts and responsiveness, and I have no further comments. It has been a pleasure to participate in the review of this interesting and high-quality work.

Open Access This Peer Review File is licensed under a Creative Commons Attribution 4.0 International License, which permits use, sharing, adaptation, distribution and reproduction in any medium or format, as long as you give appropriate credit to the original author(s) and the source, provide a link to the Creative Commons license, and indicate if changes were made.
In cases where reviewers are anonymous, credit should be given to 'Anonymous Referee' and the source.
The images or other third party material in this Peer Review File are included in the article’s Creative Commons license, unless indicated otherwise in a credit line to the material. If material is not included in the article’s Creative Commons license and your intended use is not permitted by statutory regulation or exceeds the permitted use, you will need to obtain permission directly from the copyright holder.
To view a copy of this license, visit https://creativecommons.org/licenses/by/4.0/

Response letter:

We sincerely thank all three reviewers for their constructive and insightful comments, which have significantly helped strengthen the manuscript. In the revised version, we have carefully addressed all reviewer comments and substantially expanded the experimental validation, quantitative characterization, and discussion of the device architecture and operating mechanism. Below, we provide a point-by-point response to each comment and indicate the corresponding revisions made in the manuscript and Supplementary Information. All newly added text is highlighted in red.

Reviewer #1 (Remarks to the Author):

The authors presented an interactively addressable organic metadevice platform based on the electrochemical redox modulation of conducting polymers. By conformally integrating plasmonic Au nanorod (NR) nanoantennas with a thin polyaniline (PANI) coating, the platform leveraged locally voltage-controlled redox-state changes that altered the complex refractive index and absorption of PANI, thereby enabling pixel-level binary ("on/off") modulation of the nanoantenna scattering amplitude. A planar indium tin oxide (ITO) fan-out architecture electrically isolated and independently drove each metasurface pixel in a two-dimensional array. Operating at visible wavelengths, the system demonstrated 4 x 4 and 6 x 6 independently addressable arrays, sub-volt actuation (-0.2 V / +0.6 V), and millisecond-scale switching (~24 ms and ~2 ms). Embedded in a closed-loop control scheme, user inputs from a keyboard or gamepad were translated in real time into pixel-resolved voltage vectors, enabling dynamic holographic character projection and interactive gaming, while confirming negligible electrical crosstalk, reasonably uniform pixel responses, and stable operation under evolving holographic scenes.

By elevating an electrochemically tunable metasurface from a material-level demonstration to a system-level, user-programmable interactive photonic platform, this work offered an intrinsic (non-external-SLM) route toward visible-wavelength, two-dimensional programmable wavefront control and holographic display. It further showcased software-defined voltage-pattern driving for reconfigurable content generation and a potentially large optical state space (e.g., 2^36 states for a 6 x 6 array). While pixel-level electrically addressed organic metasurfaces appeared promising for display/projection and reconfigurable photonic devices, the manuscript still benefited from stronger engineering metrics and broader validation. Overall, it constitutes a clearly novel platform-style proof of concept that can meet the high publication standards if key experiments and analyses are strengthened.

We sincerely thank the reviewer for the positive and encouraging evaluation of our work. We also appreciate the reviewer's assessment that "*it constitutes a clearly novel platform-style proof of concept*". We have carefully addressed all specific points below and believe the revisions have further strengthened the manuscript.

1. The device operation relies on a liquid electrolyte, which makes the work closer to a platform-level validation than an integrable product prototype. The authors could strengthen the manuscript by providing more data to support long-term operational stability and a clear packaging/encapsulation pathway.

OUR RESPONSE and **ACTIONS TAKEN**:

We thank the reviewer for this important suggestion. In response, we developed an encapsulated metadevice architecture and performed long-term cycling measurements. The revised manuscript now

includes detailed descriptions of the packaging strategy together with stability characterization over 1200 switching cycles, showing only a ~2% reduction in intensity contrast. These additions strengthen the system-level validation of the metadevice platform.

Page 8, Main text:

"An encapsulated metadevice can operate for over 1200 switching cycles with only a ~2% reduction in intensity contrast (Supplementary Fig. 15; Supplementary Note 4; Supplementary Fig. 16)."

Page 12, Main text:

"For an integrated prototype demonstration and quantitative evaluation of device performance, the metadevice was encapsulated using an ITO/SiO_2 substrate adhered to the metasurface substrate with a UV-curable adhesive (Norland NOA 81) containing 7-μm glass spacer beads. After UV curing, the device was infiltrated with 0.5 M HNO_3 electrolyte. The ITO layer, in contact with the electrolyte, served as the counter electrode and was connected to the grounded channel of the McDAC. The ITO fan-out electrodes were interfaced with a flat flexible ribbon cable (AWM 20624) through a custom-built sample holder, enabling independent electrical addressing of each metasurface pixel."

Supplementary Fig. 15:

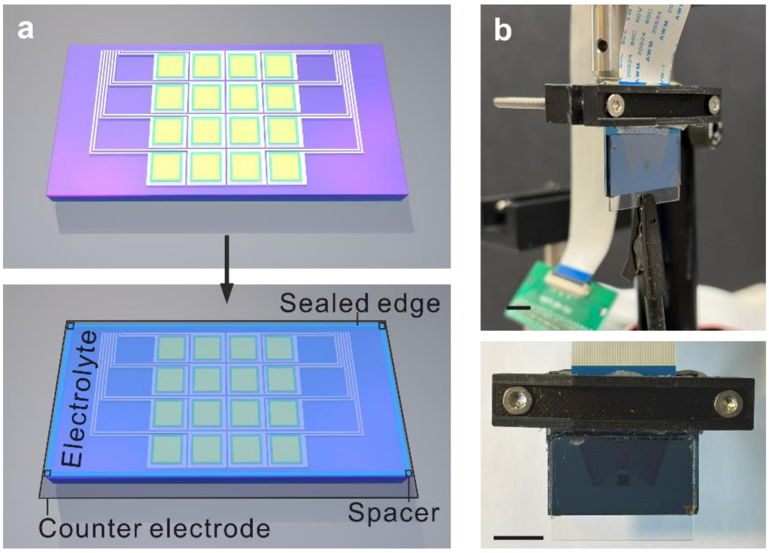

Supplementary Fig. 15 | Encapsulation of the organic metadevice. a, Schematic illustration of the metadevice encapsulation strategy. **b**, Photograph of the metadevice integrated with an ITO-glass counter electrode and electrolyte layer, mounted on a custom-built sample holder with external electrical interfacing. Scale bar, 1 cm.

Supplementary Note 4: Evaluation of operational stability

"The encapsulated metadevice maintains stable electrochemical switching over 1200 switching cycles, with only a ~2% reduction in intensity contrast (Supplementary Fig. 16), demonstrating that the switching functionality is largely preserved during repeated operation. Minor intensity drift observed over extended cycling may originate from gradual electrochemical or structural changes in the conducting polymer layer. Previous studies have demonstrated substantially longer switching lifetimes in conducting polymer systems without significant degradation[7–9], suggesting that the operational durability of the organic metadevice could be further improved through materials optimization and device engineering."

Supplementary Fig. 16:

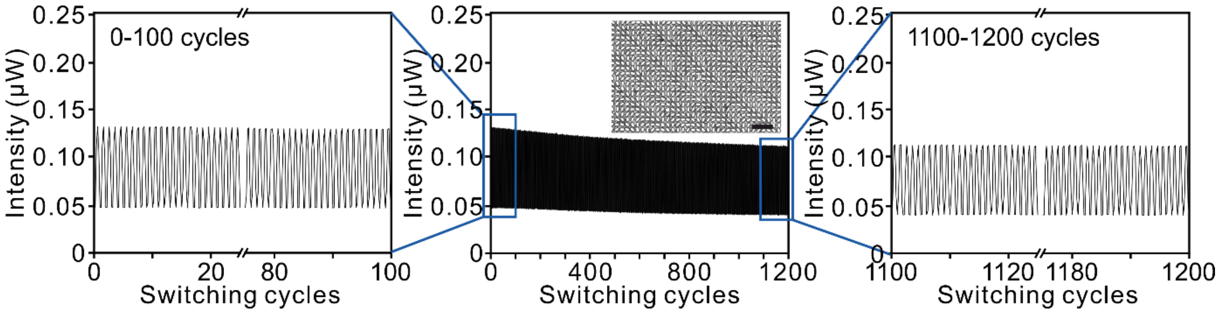

Supplementary Fig. 16 | Evaluation of operational stability. Anomalously reflected light intensity recorded from the metadevice while all pixels were simultaneously switched between -0.2 V (on) and +0.6 V (off), with each voltage applied for 2.5 s over 1200 switching cycles. The enlarged traces show representative switching cycles at the initial and final stages of the cycling test. The inset shows an SEM image of the PANI-coated AuNRs after cycling, revealing no obvious morphological degradation of the metasurface. Scale bar, 1 μm.

2. The demonstrated arrays are limited to 4 x 4 and 6 x 6, with a 350 um pixel pitch, constituting a low-resolution proof of concept. Both the scale and image fidelity are therefore constrained and remain orders of magnitude away from high-fidelity holography/display, motivating additional evidence from higher-resolution arrays and pixel-level experiments.

OUR RESPONSE and **ACTIONS TAKEN**:

We thank the reviewer for this constructive comment. The 4 × 4 and 6 × 6 arrays represent proof-of-concept demonstrations intended to establish the feasibility of independently addressable 2D organic metasurfaces operating at visible wavelengths. In the current work, the demonstrated array formats were chosen as a practical balance between fabrication throughput, routing complexity, and device yield for this first-generation laboratory-scale prototype, rather than reflecting a fundamental limitation of the addressing concept itself.

The present study establishes the foundational platform concept for interactively addressable organic metadevices and provides a basis for future development toward higher-density implementations. We have clarified this point in the revised manuscript and added a brief discussion.

Page 9, Main text:

"Scaling the 2D in-plane fan-out arrangement toward higher-density arrays will require further optimization of the routing scheme and fabrication throughput."

3. Although the authors presented switching times and intensity contrast, the performance metrics are not yet comprehensive. In particular, a systematic, closed-loop evaluation of key engineering figures of merit, such as the diffraction efficiency, power consumption, cycling lifetime, and long-term drift, are still missing, with the current discussion relying more on the qualitative descriptions of "stable operation/low crosstalk."

OUR RESPONSE and **ACTIONS TAKEN**:

We thank the reviewer for this important suggestion. Following the reviewer's suggestions, we added

quantitative evaluations of diffraction efficiency, power consumption, cycling lifetime, and long-term operational drift. Specifically, we added the following discussions to the revised manuscript and Supplementary Information.

Supplementary Note 3: Evaluation of the holographically projected pixel array

"The metadevice exhibits a diffraction efficiency of 1.2%, defined as the intensity ratio between the anomalously reflected light and the incident circularly polarized light."

Supplementary Fig. 17:

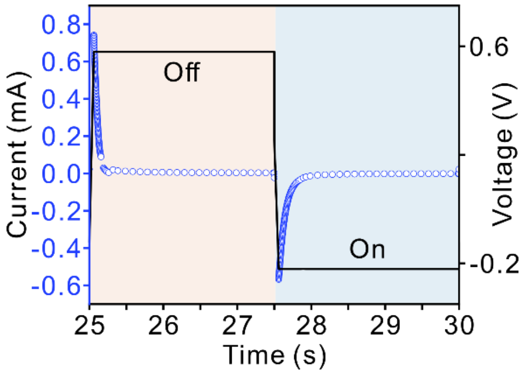

Supplementary Fig. 17 | Chronoamperometric characterization of electrochemical switching. Current response of the metadevice under alternating voltage modulation between +0.6 V (off) and -0.2 V (on), with each voltage applied for 2.5 s. The integrated current response was used to quantify the corresponding energy and power consumption during electrochemical switching. The corresponding energy and power consumption during electrochemical switching were quantified to be 1.58 mJ cm^{-2} and 0.630 μW cm^{-2} for oxidation (off), and 0.64 mJ cm^{-2} and 0.258 μW cm^{-2} for reduction (on), respectively.

We further evaluated the operational stability of the encapsulated metadevice over 1200 switching cycles (Supplementary Fig. 16, page 3 of this response letter), during which the intensity contrast decreased by only ~2%, indicating stable electrochemical switching with minimal long-term drift during repeated operation.

4. The authors employed Pancharatnam-Berry (PB) phase metasurfaces to encode holographic phase profiles via orientation control of Au NRs. However, no characterizations of orientation uniformity, scattering spectra, phase gradient fidelity, or polarization conversion efficiency are provided. The device primarily operates via amplitude modulation through PANI absorption. Is phase modulation preserved during switching? How do the potential phase distortions affect the fidelity of holographic reconstruction?

OUR RESPONSE and **ACTIONS TAKEN**:

We thank the reviewer for the insightful comments. In our metadevice, the holographic phase profile is encoded using the PB phase through orientation control of the Au nanorods, while electrochemical switching of PANI modulates the resonant scattering amplitude.

In response to the reviewer's comments regarding orientation uniformity and phase gradient fidelity, we performed a statistical analysis of the fabricated AuNR orientations from SEM images of the metasurface, confirming good agreement between the designed and fabricated orientation angles for the discrete PB

phase levels (Supplementary Fig. 5). This supports faithful implementation of the encoded PB phase gradient.

Supplementary Fig. 5:

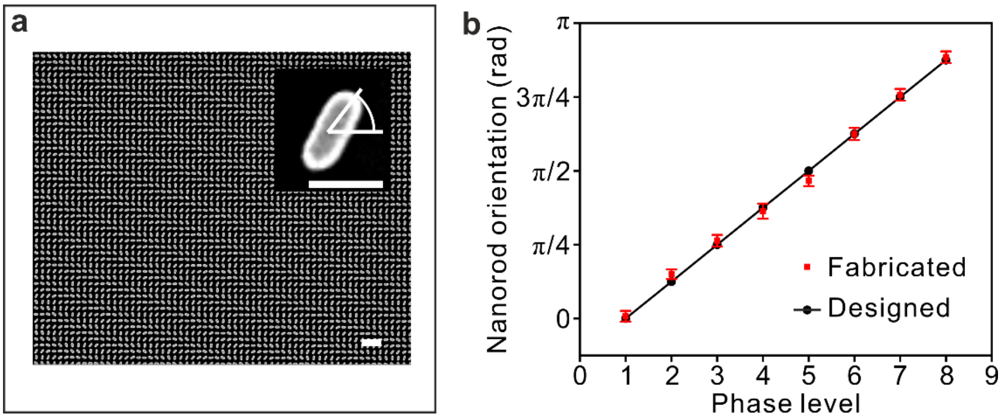

Supplementary Fig. 5 | Characterization of AuNR orientations. a, SEM image of the metasurface used for the statistical analysis of AuNR orientations. Scale bar, 1 μm. Inset: magnified SEM image of a single AuNR. Scale bar, 200 nm. **b**, Orientation uniformity analysis. The discrete orientations of AuNRs corresponding to eight PB phase levels were statistically extracted and compared with the designed values, showing good agreement with the intended phase encoding.

Also, we measured the scattering spectra of a single PANI-coated AuNR in the on and off states.

Page 5, Main text:

"In the reduced state, the PANI-coated AuNRs exhibit pronounced plasmonic scattering, whereas in the oxidized state, increased absorption suppresses scattering (Supplementary Fig. 6)."

Supplementary Fig. 6:

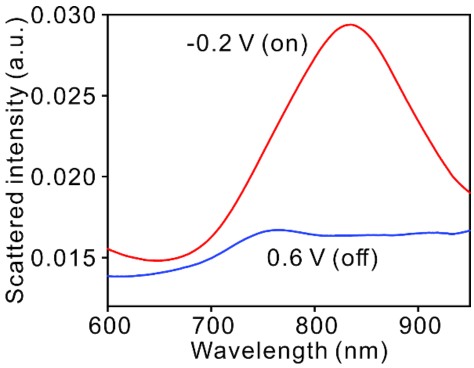

Supplementary Fig. 6 | Measured scattering spectra of a PANI-coated AuNR. In the reduced state at -0.2 V, the AuNR exhibits a plasmonic resonance (red curve), whereas in the oxidized state at +0.6 V, the resonance is strongly suppressed (blue curve).

Supplementary Note 3: Evaluation of the holographically projected pixel array

"The polarization conversion efficiency is 6%, defined as the intensity ratio between the cross-polarized reflected light and the total reflected light under circularly polarized illumination."

We further performed numerical simulations of the phase delay as a function of AuNR orientation for both reduced (on) and oxidized (off) states (Supplementary Fig. 4). The simulations show that the PB phase modulation is retained in both electrochemical states over the full orientation range. Furthermore, the experimentally reconstructed holographic patterns remain well-defined during electrochemical switching (Figs. 2–4), demonstrating that the fidelity of holographic reconstruction is preserved.

Page 5, Main text:

"The encoded PB phase profile is retained in the reduced and oxidized PANI states (Supplementary Fig. 4)."

Supplementary Fig. 4:

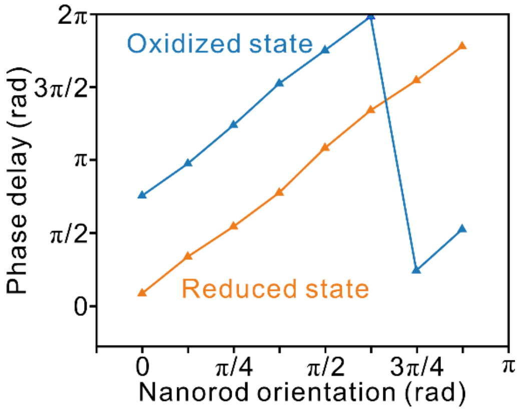

Supplementary Fig. 4 | Simulated phase profiles for reduced and oxidized PANI states. Simulated phase profiles as a function of AuNR orientation for reduced and oxidized PANI states (h = 165 nm).

5. The reasons of using circularly polarized light (CPL) should be clarified. The optical efficiency of the metadevices under CPL should be quantified. Can the devices operate under linearly polarized or unpolarized illumination?

OUR RESPONSE and **ACTIONS TAKEN**:

We thank the reviewer for this helpful suggestion. Circularly polarized illumination is required because the holographic phase profile is encoded using the PB phase, which relies on helicity-dependent geometric phase modulation. We have added the relevant references (Refs. 57-60) in the revised manuscript.

We have also added the conversion efficiency under circularly polarized illumination in the revised Supplementary Information.

Supplementary Note 3: Evaluation of the holographically projected pixel array

"The polarization conversion efficiency is 6%, defined as the intensity ratio between the cross-polarized reflected light and the total reflected light under circularly polarized illumination."

The metadevice can also operate under linearly polarized illumination, where both helicity channels are simultaneously excited, resulting in a superposition of the corresponding holographic projections (Supplementary Fig. 9). Under unpolarized illumination, similar superposed holographic patterns are expected with reduced contrast due to incoherent mixing of the two polarization channels.

Supplementary Fig. 9:

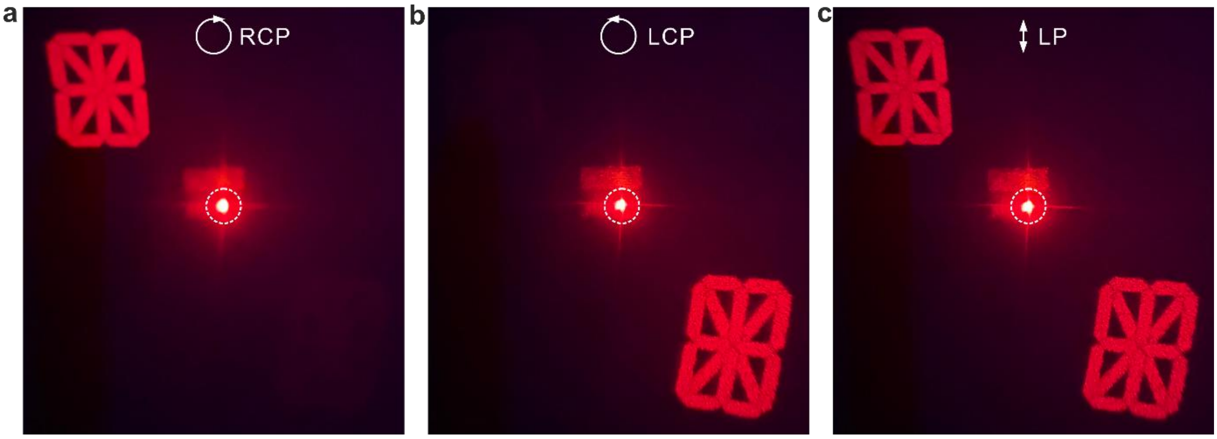

Supplementary Fig. 9 | Polarization dependence of the hologram. Experimental holographic projections under RCP, LCP, and LP illumination. Under LP illumination, both helicity channels are simultaneously excited, resulting in superposed holographic projections.

6. Supplementary Fig. 1 presents the simulated on/off contrast ratio. However, the experimentally achieved on/off contrast is lower. Please explain the origin of this gap. Can the on/off contrast ratio be improved by optimizing the PANI thickness or oxidation state?

OUR RESPONSE and **ACTIONS TAKEN**:
We thank the reviewer for this valuable comment. The lower experimentally measured on/off contrast compared with the simulated results mainly arises from the differences between the idealized simulation and real experimental geometries. In practice, both the fabricated AuNRs and electrochemically grown PANI shells inevitably exhibit structural and morphological imperfections.

We further investigated the influence of PANI thickness on the switching performance and included the new results in the revised Supplementary Information.

Page 6, Main text:
"The intensity contrast can be improved by increasing the PANI thickness, with an accompanying trade-off in switching speed (Supplementary Fig. 10)."

Supplementary Fig. 10:

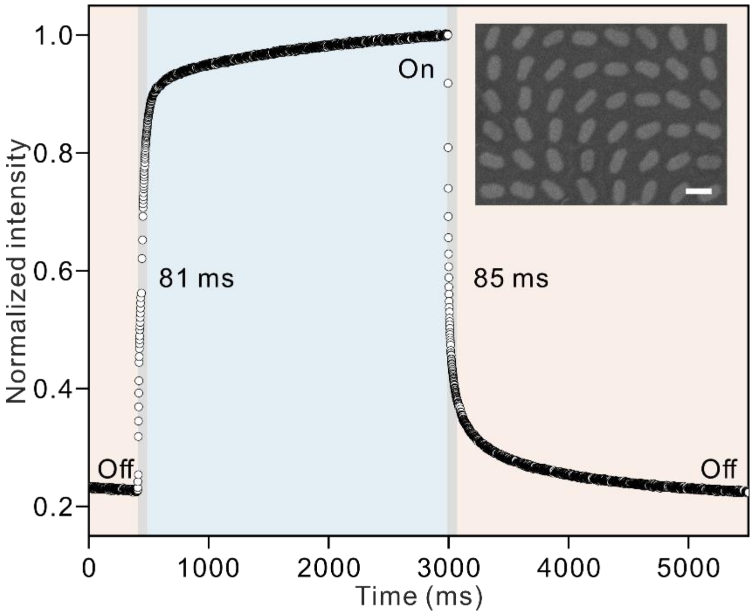

Supplementary Fig. 10 | Switching performance of the metadevice with increased PANI thickness. Temporal response of a metasurface pixel with a thicker PANI coating (250 nm) under alternating voltage modulation between -0.2 V (on) and +0.6 V (off). The thicker PANI coating improves the intensity contrast to 5.2:1, while increasing the switching times to 81 ms and 85 ms for the off-to-on and on-to-off transitions, respectively.

7. The electrochemical characteristics of the redox-driven switching of PANI such as cycling endurance and the correlation between voltage modulation and oxidation state are not provided. Basic electrochemical data should be included to support the switching mechanism.

OUR RESPONSE and **ACTIONS TAKEN**:

We thank the reviewer for this important suggestion. In response, we included cyclic voltammetry characterization of the PANI-coated metasurface (Supplementary Fig. 11a) together with voltage-dependent measurements of the anomalously reflected light intensity (Supplementary Fig. 11b). The anomalously reflected light intensity reversibly changes as the applied voltage is varied between -0.2 V and +0.6 V, confirming the correlation between the applied voltage and the redox state of PANI.

Supplementary Fig. 11:

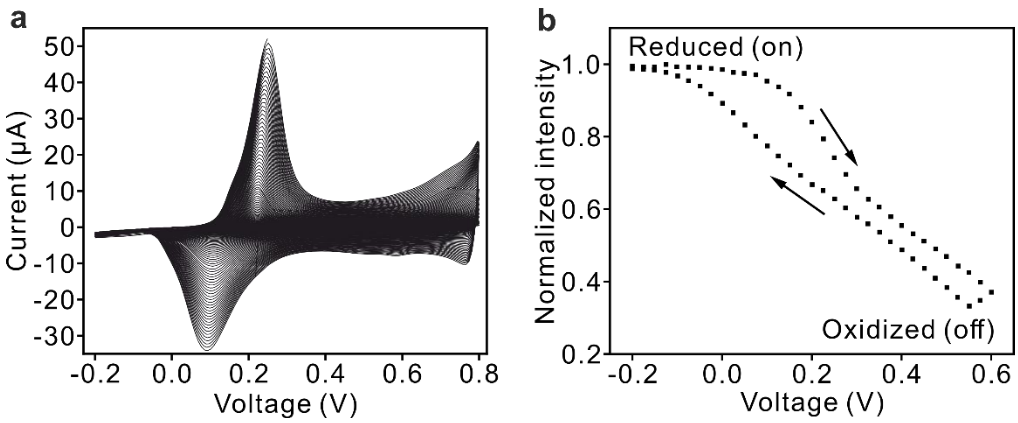

Supplementary Fig. 11 | Electrochemical characterization of the metadevice. a, Cyclic voltammetry response of the PANI-coated metasurface. **b**, Voltage-dependent anomalously reflected intensity, showing reversible switching between the reduced (on) and oxidized (off) states of PANI.

8. The authors state that negligible electrical crosstalk is achieved, yet no supporting data are presented. Quantitative crosstalk characterization such as intensity leakage into neighboring off-pixels during switching should be included.

OUR RESPONSE and **ACTIONS TAKEN**:
We thank the reviewer for this valuable comment. To quantitatively evaluate the electrical crosstalk, we sequentially switched on one target pixel at a time while keeping all other pixels in the off state, and analyzed the corresponding intensity variations across the entire 6 × 6 array. The intensity analysis under both row-by-row and column-by-column switching sequences (Supplementary Fig. 13) confirms negligible crosstalk in the independently addressable metadevice architecture. We have added the following discussion to the revised manuscript and Supplementary Information.

<u>Page 7, Main text:</u>
"By sequentially switching on one pixel while keeping all other pixels in the off state, the addressed pixel exhibits much larger intensity variation than the non-addressed neighboring pixels, confirming negligible crosstalk in the metadevice (Supplementary Fig. 13)."

<u>Supplementary Fig. 13:</u>

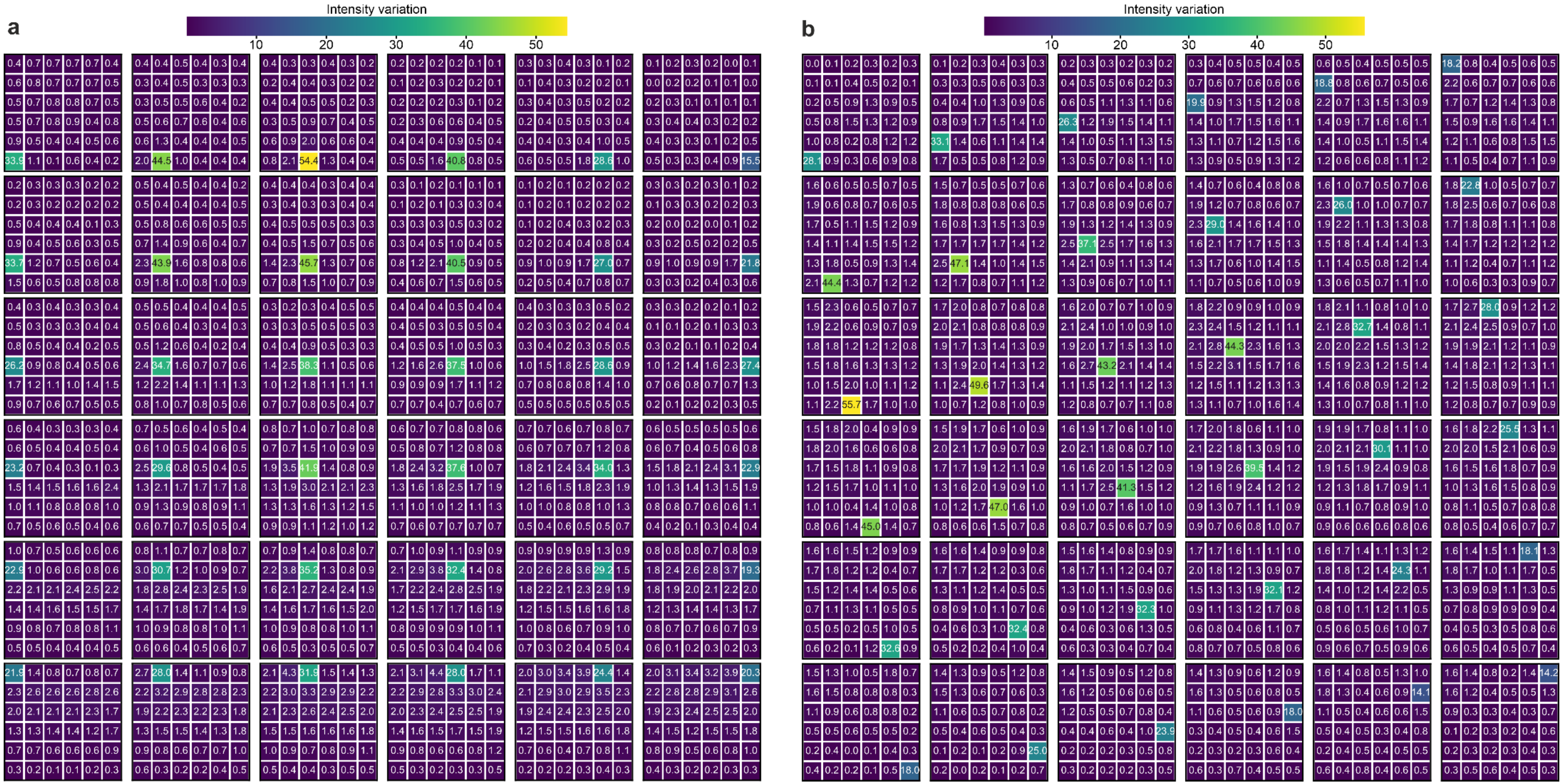

Supplementary Fig. 13 | Crosstalk evaluation of the pixel array. Intensity variations of all pixels during sequential switching of individual target pixels under row-by-row (**a**) and column-by-column (**b**) addressing sequences.

Reviewer #2 (Remarks to the Author):

This manuscript presents an interactively addressable 2D organic metadevice based on electrochemical modulation. The authors demonstrate a platform where ultrathin polyaniline (PANI) is conformally integrated with plasmonic gold nanorods (AuNRs) to create metasurface pixels that can be switched independently. By employing a planar fan-out ITO electrode architecture, the system achieves independent 2D pixel control without the crosstalk typical of liquid-crystal systems. The interdisciplinary nature of the work—combining micro-nanofabrication, polymer chemistry, and software-defined control—is well-suited for the broad readership of Nature Communications. Due to several issues, I recommend a revision of this manuscript.

We sincerely thank the reviewer for the thoughtful and constructive evaluation. We greatly appreciate the reviewer's recognition that our manuscript presents "*the interdisciplinary nature of the work—combining micro-nanofabrication, polymer chemistry, and software-defined control—is well-suited for the broad readership of Nature Communications*."

1. About the scalability and the Definition of "High-Density". The authors emphasize that this platform paves the way for "high-density" addressable 2D organic meta-optics. The current demonstration is limited to 4*4 and 6*6 arrays using a planar fan-out routing scheme. As the pixel count scales towards megapixels (or even 32*32), the real estate required for individual ITO leads in a single plane will inevitably lead to a drop in the optical fill factor or an unmanageable increase in chip footprint. Can the authors provide a quantitative analysis of the physical scaling limits of this fan-out architecture? Calling the current scheme a route to "high-density" requires a more rigorous boundary definition.

OUR RESPONSE and **ACTIONS TAKEN**:
We thank the reviewer for this constructive comment. The 4 × 4 and 6 × 6 arrays represent proof-of-concept demonstrations intended to establish the feasibility of independently addressable 2D organic metasurfaces operating at visible wavelengths. In the current work, the demonstrated array formats were chosen as a practical balance between fabrication throughput, routing complexity, and device yield for this first-generation laboratory-scale prototype, rather than reflecting a fundamental limitation of the addressing concept itself.

Scaling the single-plane fan-out architecture toward substantially larger pixel counts will require careful consideration of routing density and chip footprint. Using the current routing parameters of 350 μm pixel pitch, 15 μm ITO linewidth, and 10 μm spacing, a simple routing estimate indicates that the present single-plane fan-out architecture is practically extendable to arrays on the order of tens-by-tens of pixels before the effective outer-pixel area becomes strongly constrained. Further scaling beyond this regime would require higher-resolution routing, improved device integration, or alternative addressing architectures.

Accordingly, we moderated the wording related to "high-density" in the revised manuscript and retained it only in the context of future perspectives. The present study establishes the foundational platform concept for interactively addressable organic metadevices and provides a basis for future development toward higher-density implementations. We have clarified this point in the revised manuscript and added a brief discussion.

Page 9, Main text:

"Scaling the 2D in-plane fan-out arrangement toward higher-density arrays will require further optimization of the routing scheme and fabrication throughput."

2. The system currently operates within an electrochemical cell filled with 0.5M~HNO3. While acceptable for a proof-of-concept, a "user-interactive" device operating in a strong acid environment faces severe challenges regarding packaging, component corrosion, and portability. Have the authors conducted any durability tests on the electrodes and AuNRs under prolonged exposure to this acidic environment? The authors mention solid-state electrolytes as a future direction; however, if the ion diffusion rate drops significantly in a solid matrix, would the "millisecond-scale" switching still be achievable for real-time interaction?

OUR RESPONSE and **ACTIONS TAKEN**:

We greatly appreciate the reviewer's comments. To evaluate device durability in the acidic electrolyte environment, we performed long-term switching measurements on encapsulated metadevices operated in 0.5 M HNO_3 electrolyte. The metadevice maintained stable switching behavior over 1200 cycles with only a ~2% decrease in intensity contrast (Supplementary Fig. 16). In addition, SEM characterization after repeated switching cycles showed no obvious structural and morphological degradation of the AuNR metasurface. We have included the durability evaluation in the revised Supplementary Information.

Supplementary Fig. 16:

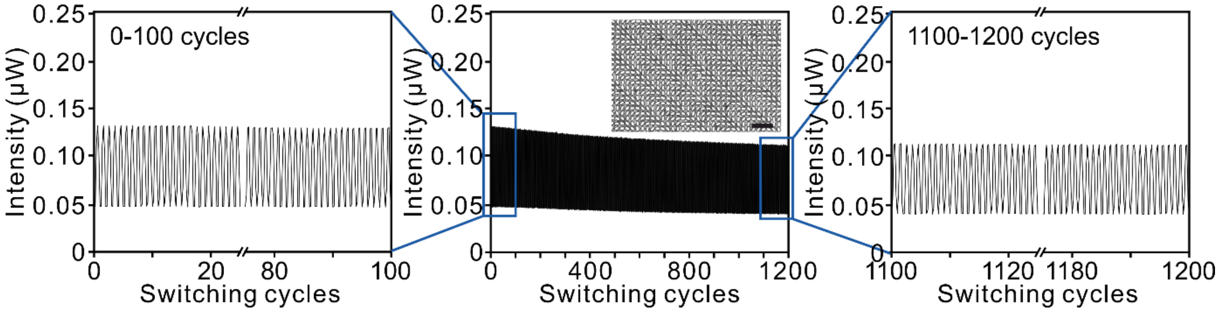

Supplementary Fig. 16 | Evaluation of operational stability. Anomalously reflected light intensity recorded from the metadevice while all pixels were simultaneously switched between -0.2 V (on) and +0.6 V (off), with each voltage applied for 2.5 s over 1200 switching cycles. The enlarged traces show representative switching cycles at the initial and final stages of the cycling test. The inset shows an SEM image of the PANI-coated AuNRs after cycling, revealing no obvious morphological degradation of the metasurface. Scale bar, 1 μm.

Regarding future solid-state or gel-electrolyte implementations, reduced ion mobility may lead to slower electrochemical response compared with liquid electrolytes. Nevertheless, previous electrochromic and conducting-polymer devices using gel electrolytes have demonstrated dynamic optical modulation at video-rate frequencies (Ref. 36). Therefore, solid-state electrolyte integration represents a promising future direction and may further motivate optimization of ion transport and device architecture for high-speed switching performance. We have added a brief discussion in the revised manuscript.

Page 9, Main text:

"Future implementation using gel or solid-state electrolytes may improve device portability and environmental robustness, with further optimization of ion transport and switching speed[36, 67-69]."

3. The reported intensity contrast is approximately 3.5:1. What is the fundamental physical bottleneck for this relatively low contrast? Is it residual scattering in the oxidized PANI state or insufficient extinction of the plasmonic resonance? In the 6*6 array, did the authors observe any optical crosstalk? Specifically, when adjacent pixels are in different "On/Off" states, does the diffractive overlap affect the fidelity of the reconstructed holographic image?

OUR RESPONSE and **ACTIONS TAKEN**:

We thank the reviewer for these insightful comments. In practice, structural imperfections in the fabricated AuNRs and electrochemically grown PANI shells can lead to residual scattering under the oxidized state, thereby reducing the experimentally observed contrast ratio.

To quantitatively evaluate the crosstalk, we sequentially switched on one target pixel at a time while keeping all other pixels in the off state, and analyzed the corresponding intensity variations across the entire 6 × 6 array. The intensity analysis under both row-by-row and column-by-column switching sequences (Supplementary Fig. 13) confirms negligible crosstalk in the independently addressable metadevice architecture. No obvious degradation of holographic fidelity was observed when adjacent pixels were switched between different on/off states, indicating that diffractive overlap between neighboring pixels remains minimal under the current operating conditions. We have added the following discussion to the revised manuscript and Supplementary Information.

Page 7, Main text:

"By sequentially switching on one pixel while keeping all other pixels in the off state, the addressed pixel exhibits much larger intensity variation than the non-addressed neighboring pixels, confirming negligible crosstalk in the metadevice (Supplementary Fig. 13)."

Supplementary Fig. 13:

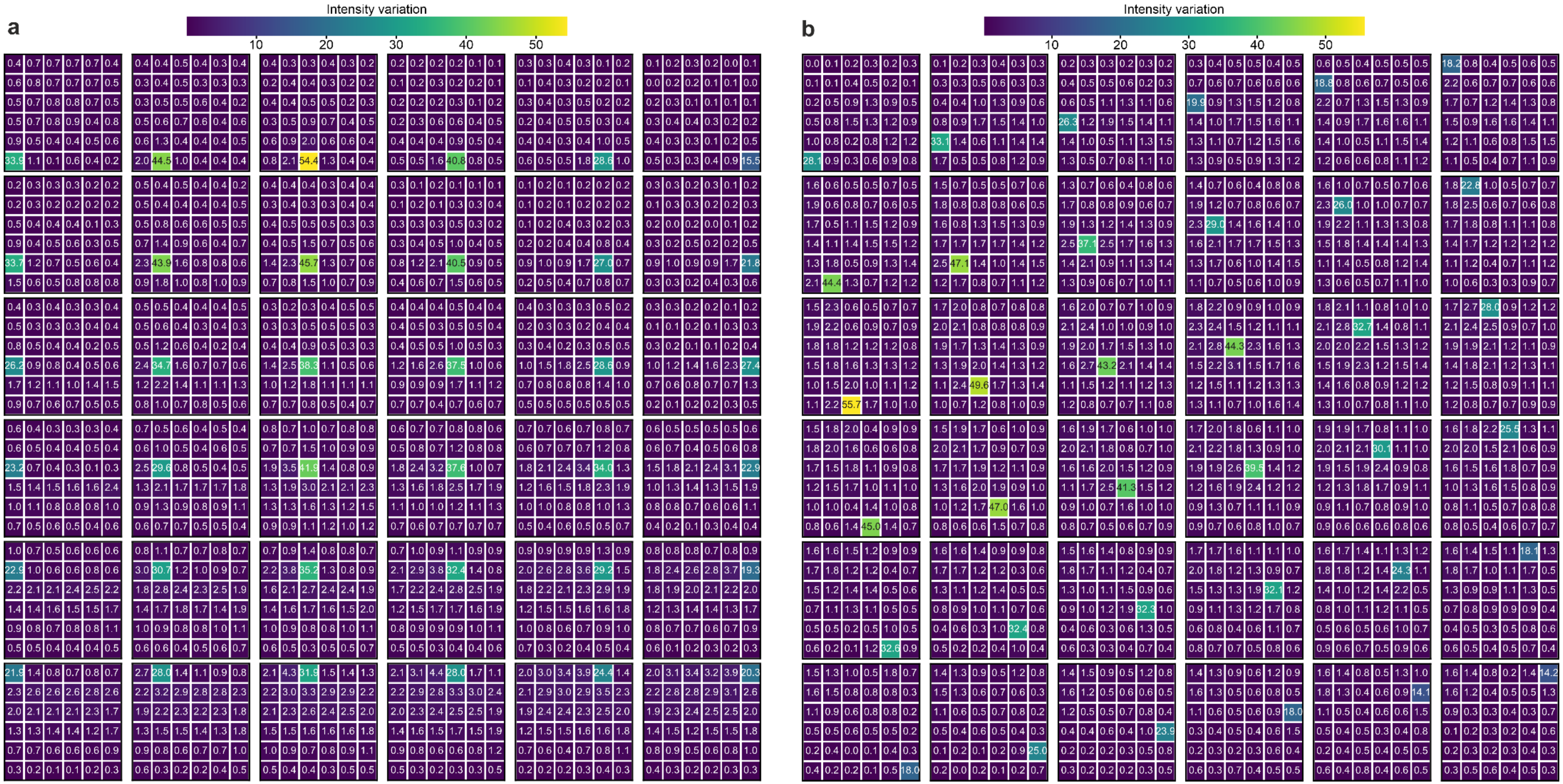

Supplementary Fig. 13 | Crosstalk evaluation of the pixel array. Intensity variations of all pixels during sequential switching of individual target pixels under row-by-row (**a**) and column-by-column (**b**) addressing sequences.

4. About Electrochemical Stability and Cycle Life. Redox-active polymers often undergo volume changes during cycling, which can lead to delamination from metallic nanostructures. Did the authors perform long-term cycling tests (e.g., >1,000 or 10,000 cycles)? Please provide data or SEM imagery of the PANI-coated AuNRs after extensive switching to confirm the structural and functional integrity of the conformal coating.

OUR RESPONSE and **ACTIONS TAKEN**:
We appreciate the reviewer raising this point, which is closely related to question 2 (page 12 of this response letter). We performed long-term switching measurements of the metadevice, demonstrating stable switching behavior over 1200 cycles (Supplementary Fig. 16). In the same figure, we have also included an SEM image of the PANI-coated AuNRs after cycling, revealing no obvious morphological degradation or delamination of the metasurface.

Some references should be included in the manuscript:
① Li J, Yu P, Zhang S, et al. A reusable metasurface template[J]. Nano letters, 2020, 20(9): 6845-6851.
② Fan, X., Xiong, W., Xu, K. et al. Spatial light modulator via optically addressed metasurface. Nat. Nanotechnol. (2026). https://doi.org/10.1038/s41565-026-02128-x
③ Li X, Chen QM, Zhang X, Zhao RZ, Xiao SM et al. Time-sequential color code division multiplexing holographic display with metasurface. Opto-Electron Adv 6, 220060 (2023). DOI: 10.29026/oea.2023.220060

OUR RESPONSE and **ACTIONS TAKEN**:
We thank the reviewer for the helpful suggestions. We have added the relevant literature as Refs. 23 and 27–28 in the revised manuscript.

Page 2, Main text:
"A recent work by Fan *et al*. demonstrated an optically addressed metasurface SLM[28]."

Reviewer #3 (Remarks to the Author):

What are the noteworthy results?
This study demonstrates a significant breakthrough in active metasurfaces by implementing a planar fan-out architecture that enables independent, pixel-level addressing of PANI-integrated plasmonic nanoantennas in the visible regime. The device achieves millisecond-scale switching dynamics while maintaining negligible pixel-to-pixel crosstalk. The practical utility of this platform is further validated by a user-driven, interactive holographic system capable of real-time rendering and interactive gaming, highlighting its potential for programmable, responsive photonic applications.

We sincerely thank the reviewer for the constructive feedback and for the positive remark that our manuscript constitutes "*a significant breakthrough in active metasurfaces*".

Will the work be of significance to the field and related fields? How does it compare to the established literature? If the work is not original, please provide relevant references.
While the manuscript demonstrates an integrated platform for interactive holographic displays, the overall conceptual novelty and scientific contribution appear somewhat limited for a journal of Nature Communications' standing. The work feels more like an incremental engineering integration of established materials and electronic designs rather than a fundamental breakthrough. My concerns are detailed as follows:
1. Incremental Material Innovation
The use of PANI-coated Gold Nanorods (AuNRs) as the active medium is not a novel structural or material advancement. Similar electrochemical modulation mechanisms and material architectures have already been extensively explored for electrochromic and plasmonic display applications (e.g., https://www.science.org/doi/10.1126/sciadv.aaw2205).The authors fail to clearly differentiate how their specific implementation provides a superior or fundamentally different optical modulation mechanism compared to these well-established benchmarks.
2. Limited Advancement in Electronic Engineering
The electronic control strategy, while functional, follows conventional engineering practices.
• Addressing Scheme: Using a 16-pixel array for alphanumeric rendering is a standard approach in display technology and does not represent a significant leap in pixel density or addressing logic for metasurfaces.
• Integration: The methodology of integrating electrochemically active soft materials onto electronic controllers for individual pixel modulation has been previously demonstrated in similar contexts (e.g., https://pubs.rsc.org/en/content/articlehtml/2024/lf/d3lf00266g). The "planar fan-out" architecture presented here appears to be a practical optimization rather than a transformative engineering principle.

OUR RESPONSE and **ACTIONS TAKEN**:
We sincerely thank the reviewer for the thoughtful comments. We would like to address these two points together, as they are closely related. The two papers cited by the reviewer belong to substantially different classes of electro-optical systems. Ref. 63 (Sci. Adv. 2019, 5, eaaw2205) focused on electrochromically tunable plasmonic nanopixels and dynamic color modulation based on PANI-coated nanoparticles, whereas Ref. 64 (RSC Appl. Interfaces 2024, 1, 719–727) demonstrated TFT-addressed electrochromic color displays based on Fabry–Pérot structures. Neither work focused on programmable

metasurface optics, holographic wavefront reconstruction, or interactive metadevice operation in the visible regime.

In contrast, our work establishes an interactively addressable organic metadevice platform that combines independently programmable metasurface pixels, PB-phase holographic wavefront engineering, electrochemical modulation, and user-interactive optical functionality within a unified architecture. Rather than focusing on electrochromic color modulation or display-oriented architectures, our study demonstrates a dynamically programmable organic metasurface platform for interactive holographic meta-optics. To further clarify these distinctions, we have added Supplementary Table 2 comparing the scope and functionality of our work with these two studies.

Page 9, Main text:

"In this work, we have demonstrated interactively addressable organic metadevices that integrate electrochemical modulation of nanoscale antennas with electronically programmable, user-driven holographic functionality. This capability is distinct from prior electrochromic plasmonic systems, which primarily focused on plasmonic color or brightness modulation, or active-matrix display addressability without dynamic wavefront engineering (Supplementary Table 2)[63,64]."

Supplementary Table 2: Comparison of the present work with representative electrochromic plasmonic systems

Aspect	This work	Ref. 63 (Sci. Adv. 2019, 5, eaaw2205)	Ref. 64 (RSC Appl. Interfaces 2024, 1, 719–727)
**System type**	Interactively addressable organic metadevice	Electrochromic plasmonic nanopixels	TFT-addressed electrochromic display
**Nanophotonic structure**	PB-phase metasurface based on PANI-coated AuNRs	PANI-coated Au nanoparticles	Fabry–Pérot electrochromic cavities
**Structural organization**	Lithographically defined metasurface pixels	Self-assembled plasmonic nanoparticle assemblies	TFT-integrated electrochromic structures
**Demonstrated architecture**	Independently addressable metasurface array	Uniformly modulated electrochromic plasmonic surface	TFT-addressed electrochromic display
**Optical functionality**	Dynamic holographic projection	Plasmonic coloration	Reflective color display
**Holographic wavefront engineering**	Yes	No	No
**Phase modulation**	Yes	No	No
**Interactive optical operation**	Yes	No	No

**Demonstrated functionality**	Interactive holographic projection and user-interactive operation	Plasmonic color switching	Active-matrix reflective color display
**Primary research focus**	Interactively programmable visible-wavelength metadevices	Electrochromic plasmonic coloration	Electrochromic display engineering

3. Disparity Between Engineering Application and Scientific Depth

The primary strength of the paper lies in its system-level application, specifically the integration with external peripherals for interactive gaming and real-time displays. However, this is largely an engineering effort. In its current form, the manuscript lacks a rigorous exploration of the underlying physics—such as the complex electrochemical-optical coupling dynamics at the organic-inorganic interface or the limits of phase/amplitude control in such hybrid systems. For a high-impact multidisciplinary journal, the scientific content feels "thin" compared to the technical implementation of the user interface.

OUR RESPONSE and **ACTIONS TAKEN**:

We appreciate the reviewer's comments. Nature Communications is a broad multidisciplinary journal in which both fundamental studies of physical mechanisms and platform-level advances that integrate materials, device architectures, and system functionality are within the scope of the journal. This perspective is also reflected in the comment of Reviewer 2 on our work: "*The interdisciplinary nature of the work—combining micro-nanofabrication, polymer chemistry, and software-defined control—is well-suited for the broad readership of Nature Communications*."

Following the reviewer's suggestions, we have further strengthened the discussion of the underlying electrochemical–optical coupling mechanisms in the revised manuscript and Supplementary Information. We included additional information on finite-element simulations of anomalous reflection intensity and electric-field distributions in reduced and oxidized PANI states, clarifying how electrochemical modulation of the PANI shell modifies plasmonic scattering in the organic metasurface system (Supplementary Note 2; Supplementary Figs. 2–3).

Page 4, Main text:

"Finite-element simulations elucidating the dependence of anomalous reflection intensity on AuNR geometry and electrochemical state of PANI are presented in Supplementary Note 2 and Supplementary Figs. 2–3."

Supplementary Note 2: Numerical simulations of organic metasurfaces

"Finite-element simulations of the anomalous reflection intensity from the organic metasurfaces were carried out using COMSOL Multiphysics. Periodic boundary conditions, waveguide ports, and perfectly matched layers were applied. The AuNR was placed on a Si/SiO_2 (100 nm)/ITO (30 nm) substrate with a periodicity of 300 nm in both the *x*- and *y*- directions. Normally incident circularly polarized light (CPL) at 633 nm was defined with an electric field ($E_x = 1$, $E_y = i$, $E_z = 0$) and cross-polarized reflectance was recorded as the anomalous reflection intensity. The dielectric function of Au was taken from Johnson and Christy[1]. At 633 nm, the complex refractive indices were set to Si ($n = 3.88$, $k = 0.02$)[2], SiO_2 ($n = 1.5$, $k = 0$), ITO ($n = 1.78$, $k = 0.003$)[3], and the aqueous electrolyte ($n = 1.33$, $k = 0$). The refractive

indices of PANI were set to n = 1.63, k = 0 in the reduced state and n = 1.10, k = 0.27 in the oxidized state, respectively[4, 5].

The simulations support binary on/off modulation of anomalous reflection intensity between the reduced and oxidized states (Supplementary Fig. 2 a,b). The fabricated AuNR dimensions reported in the main text fall within the optimized parameter region identified by the simulations. Using the AuNR geometry (220 nm × 110 nm × 50 nm), we constructed a shell-like coating model to represent localized and conformal PANI growth around individual AuNRs, with a lateral shell thickness of 75 nm. We then simulated the anomalous reflection intensity and intensity contrast between the two redox states as a function of the total structural height h, which includes the 50 nm AuNR height and the vertical PANI thickness (Supplementary Fig. 2c). The intensity contrast was defined as $I_{reduced}/I_{oxidized}$, where $I_{reduced}$ and $I_{oxidized}$ denote the anomalous reflection intensities in the reduced (on) and oxidized (off) states, respectively. The simulated contrast increases with increasing h. However, excessively thick PANI coatings may introduce delamination and slower electrochemical switching. Balancing optical performance and fabrication considerations, h = 120-165 nm was selected, corresponding to simulated contrast ratios of 2.2:1 to 3.3:1.

Simulated electric-field distributions of the PANI-coated AuNR (h = 165 nm) further support the conclusion that redox transitions of PANI modulate the plasmonic resonance and scattering behavior of the AuNRs. As shown in Supplementary Fig. 3, a large portion of the electric field extends outside the AuNR and dissipates within the surrounding dielectric environment, namely, the PANI shell. In the reduced state, the PANI-coated AuNRs exhibit pronounced plasmonic scattering, whereas in the oxidized state, increased absorption in the PANI shell suppresses the scattering intensity."

Supplementary Fig. 2:

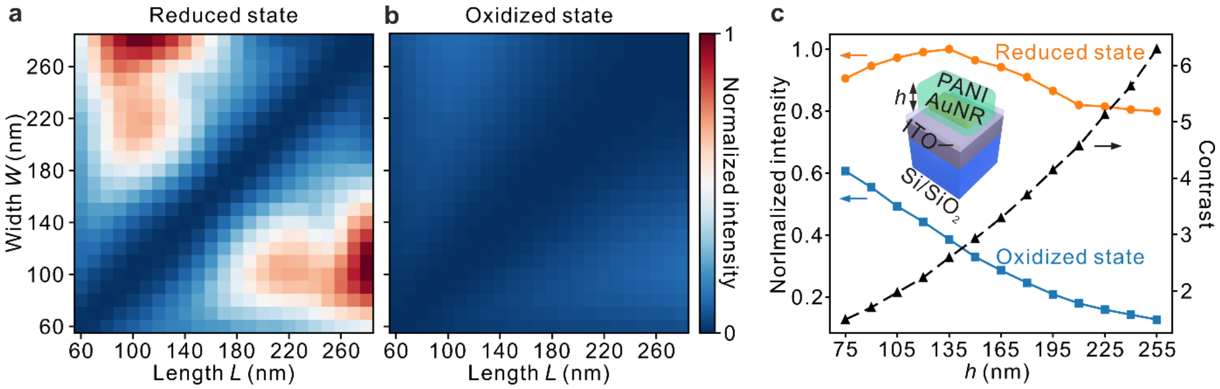

Supplementary Fig. 2 | Numerical simulations of organic metasurfaces. a,b, Simulated anomalous reflection intensity as a function of AuNR length L and width W (height fixed at 50 nm) for PANI-coated AuNRs with h = 165 nm in the reduced (**a**) and oxidized (**b**) states. Intensities are normalized to the maximum value. **c**, Simulated anomalous reflection intensity (left axis) and intensity contrast (right axis) as a function of h. The inset shows a schematic of the organic metasurface unit cell.

Supplementary Fig. 3:

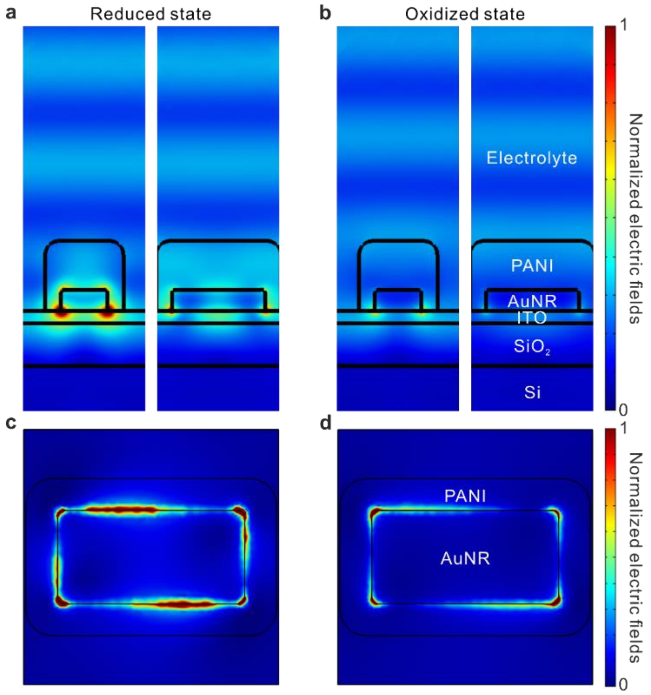

Supplementary Fig. 3 | Simulated electric-field distributions of organic metasurfaces. a,**b**, Electric-field distributions in two orthogonal cross-sectional planes through the center of the AuNR for the reduced (**a**) and oxidized (**b**) states. **c**,**d**, In-plane electric-field distributions of the AuNR for the reduced (**c**) and oxidized (**d**) states. The AuNR (220 nm × 110 nm × 50 nm) was coated with PANI with a lateral shell thickness of 75 nm and h = 165 nm.

Furthermore, we included phase-delay simulations (Supplementary Fig. 4) and measured scattering spectra of individual PANI-coated AuNRs (Supplementary Fig. 6) in the oxidized and reduced PANI states.

Page 5, Main text:

"The encoded PB phase profile is retained in the reduced and oxidized PANI states (Supplementary Fig. 4). In the reduced state, the PANI-coated AuNRs exhibit pronounced plasmonic scattering, whereas in the oxidized state, increased absorption suppresses scattering (Supplementary Fig. 6)."

Supplementary Fig. 4:

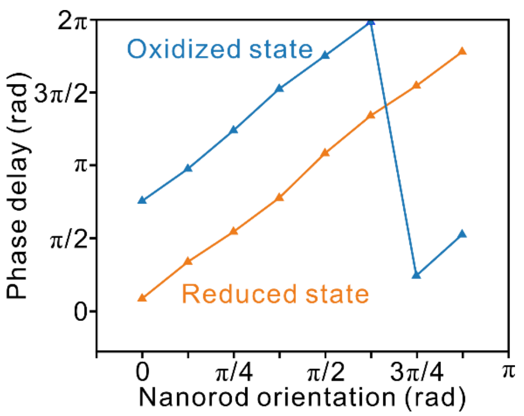

Supplementary Fig. 4 | Simulated phase profiles for reduced and oxidized PANI states. Simulated phase profiles as a function of AuNR orientation for reduced and oxidized PANI states (h = 165 nm).

Supplementary Fig. 6:

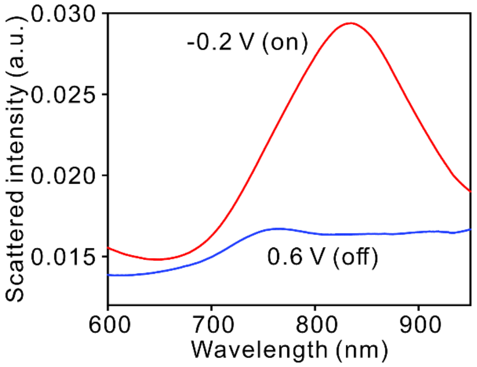

Supplementary Fig. 6 | Measured scattering spectra of a PANI-coated AuNR. In the reduced state at -0.2 V, the AuNR exhibits a plasmonic resonance (red curve), whereas in the oxidized state at +0.6 V, the resonance is strongly suppressed (blue curve).

Does the work support the conclusions and claims, or is additional evidence needed?
Generally, the experimental results align with the primary claims. However, the assertions regarding "negligible electrical crosstalk" and "stable operation" require more rigorous quantitative validation to be fully convincing.

OUR RESPONSE and **ACTIONS TAKEN**:
We thank the reviewer for this constructive comment. In response, we have added quantitative characterizations of both electrical crosstalk (Supplementary Fig. 13) and operational stability (Supplementary Fig. 16) in the revised manuscript and Supplementary Information.

Page 7, Main text:
"By sequentially switching on one pixel while keeping all other pixels in the off state, the addressed pixel exhibits much larger intensity variation than the non-addressed neighboring pixels, confirming negligible crosstalk in the metadevice (Supplementary Fig. 13)."

Supplementary Fig. 13:

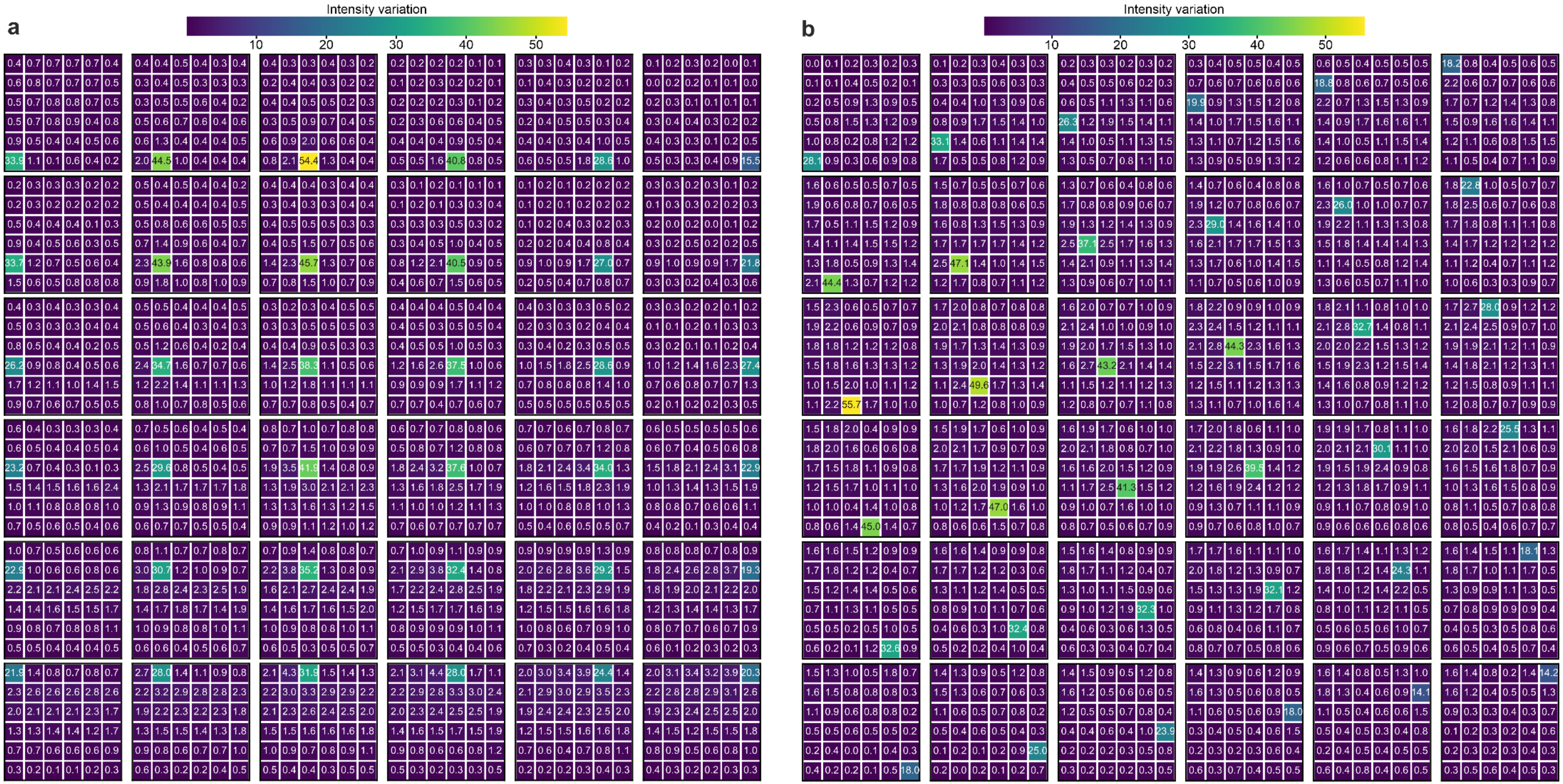

Supplementary Fig. 13 | Crosstalk evaluation of the pixel array. Intensity variations of all pixels during sequential switching of individual target pixels under row-by-row (**a**) and column-by-column (**b**) addressing sequences.

Supplementary Fig. 16:

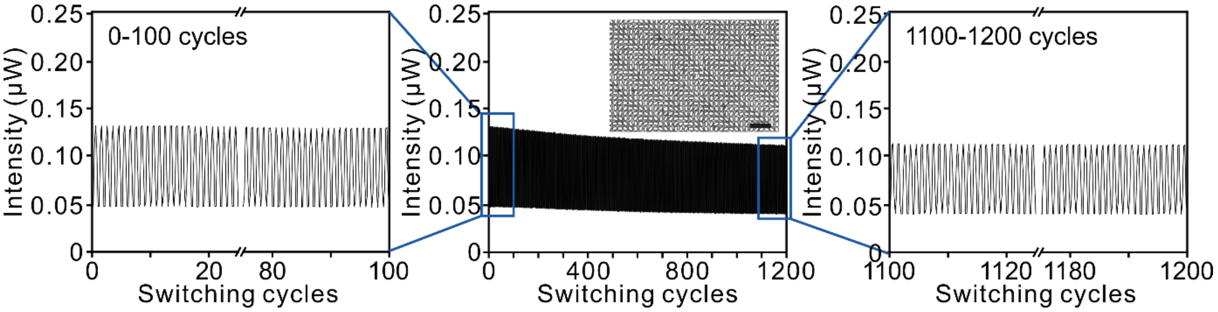

Supplementary Fig. 16 | Evaluation of operational stability. Anomalously reflected light intensity recorded from the metadevice while all pixels were simultaneously switched between -0.2 V (on) and +0.6 V (off), with each voltage applied for 2.5 s over 1200 switching cycles. The enlarged traces show representative switching cycles at the initial and final stages of the cycling test. The inset shows an SEM image of the PANI-coated AuNRs after cycling, revealing no obvious morphological degradation of the metasurface. Scale bar, 1 μm.

Are there any flaws in the data analysis, interpretation and conclusions? Do these prohibit publication or require revision?
While the conceptual demonstration is interesting, several critical gaps in the experimental evidence and technical descriptions must be addressed. These issues, ranging from fabrication precision to system integration, currently limit the reproducibility and scientific rigor of the work.
1. Ambiguity in Pixel Fabrication and Isolation:
Although the Methods section mentions EBL and PMMA masking, the Main Text lacks a visual demonstration of this critical integration. Notably, in Figure 2d, there appear to be filamentous structures at the edges of the pixels; it is unclear whether these are metallic residues or PMMA resist remnants. To substantiate the claim of negligible crosstalk, the authors should provide a step-by-step fabrication schematic and images showing the precise confinement of PANI within the pixel boundaries. This is essential to confirm that no parasitic conductive paths are formed by overgrowth or fabrication artifacts.

OUR RESPONSE and **ACTIONS TAKEN**:
We thank the reviewer for this insightful comment. In response, we added a step-by-step fabrication schematic together with corresponding optical microscopy images in the revised Supplementary Information.

Page 4, Main text:
"Detailed fabrication procedures are provided in Supplementary Note 1 and Supplementary Fig. 1."

Supplementary Note 1: Fabrication process of interactively addressable organic metadevices
"A schematic illustration of the step-by-step fabrication process of the organic metadevices is shown in Supplementary Fig. 1a. Indium tin oxide (ITO) fan-out electrodes and coarse alignment markers were fabricated by direct laser writing followed by reactive ion etching (RIE). Gold nanorod (AuNR) arrays and fine alignment markers were subsequently defined by electron-beam lithography (EBL) using poly(methyl methacrylate) (PMMA) resist, followed by Au deposition and lift-off (i–ii). To suppress pixel crosstalk during electrochemical polymerization, PMMA partition masks were defined in an additional

EBL step using the fine alignment markers (iii). The PMMA masks spatially confine electrolyte access and thereby enable localized PANI deposition on the AuNR metasurface pixels (iv). After electrochemical polymerization, the PMMA masks were removed prior to device packaging."

Supplementary Fig. 1:

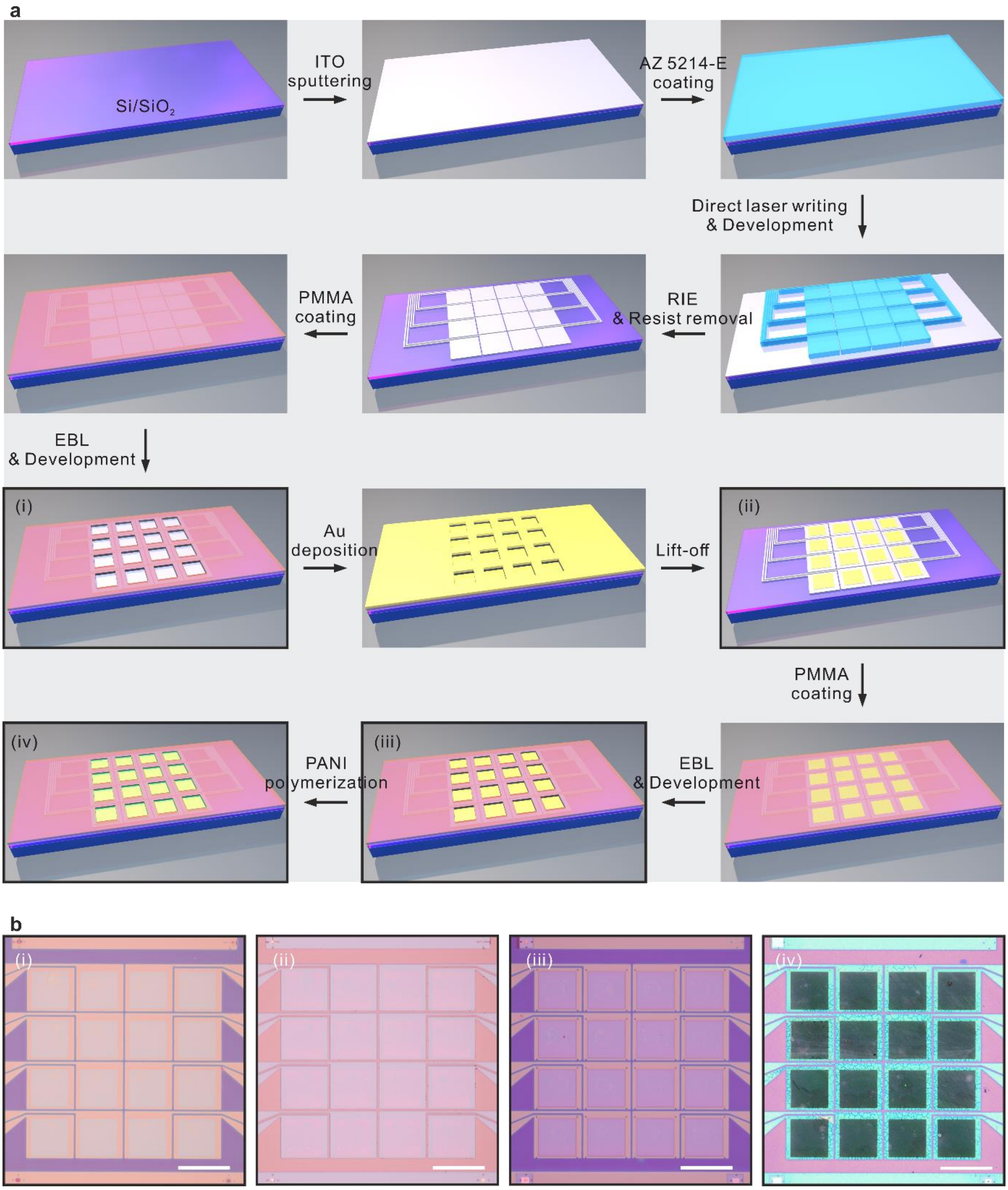

Supplementary Fig. 1 | Fabrication process of interactively addressable organic metadevices. a, Schematic illustration of the step-by-step fabrication process. **b**, Optical microscopy images of the metasurface array at fabrication steps (i)–(iv) shown in (**a**). Scale bar, 350 μm.

Furthermore, the filamentous structures observed at the corners and edges of the pixels in Fig. 2d and Supplementary Fig. 1b (iv) originate from residual PMMA mask features generated during electrochemical polymerization. These features are electrically insulating and do not form parasitic conductive pathways between neighboring pixels.

2. Incomplete Electrochemical System Integration:
The electrochemical control mechanism is central to this work, yet its integration remains underspecified. While Figure 2c illustrates the interface between the control system and the display, the physical integration of the electrochemical cell (e.g., electrolyte containment, counter/reference electrode placement within the 2D array) is not clearly explained. This omission raises significant concerns regarding the practical feasibility and scalability of the "integrated" device.

OUR RESPONSE and **ACTIONS TAKEN**:
Following the reviewer's valuable suggestion, we added additional descriptions and packaging schematics clarifying the physical integration of the electrochemical control system in the revised manuscript and Supplementary Information.

Page 12, Main text:
"For an integrated prototype demonstration and quantitative evaluation of device performance, the metadevice was encapsulated using an ITO/SiO_2 substrate adhered to the metasurface substrate with a UV-curable adhesive (Norland NOA 81) containing 7-μm glass spacer beads. After UV curing, the device was infiltrated with 0.5 M HNO_3 electrolyte. The ITO layer, in contact with the electrolyte, served as the counter electrode and was connected to the grounded channel of the McDAC. The ITO fan-out electrodes were interfaced with a flat flexible ribbon cable (AWM 20624) through a custom-built sample holder, enabling independent electrical addressing of each metasurface pixel."

Supplementary Fig. 15:

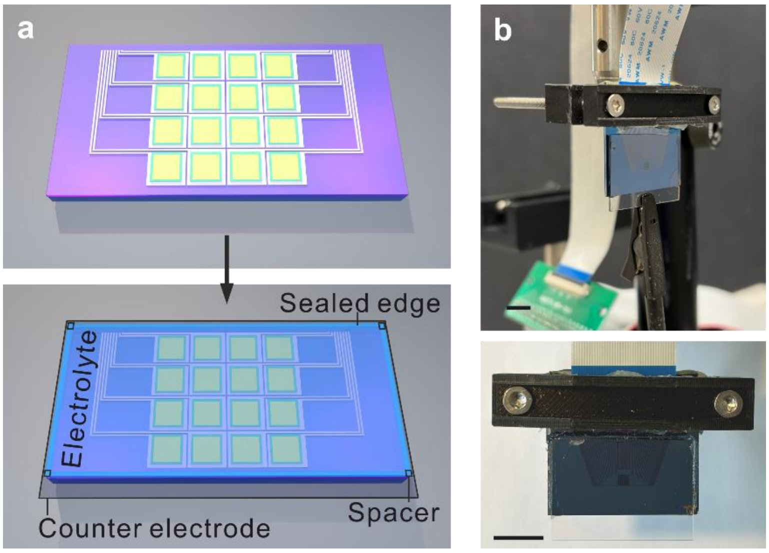

Supplementary Fig. 15 | Encapsulation of the organic metadevice. a, Schematic illustration of the metadevice encapsulation strategy. **b**, Photograph of the metadevice integrated with an ITO-glass counter electrode and electrolyte layer, mounted on a custom-built sample holder with external electrical interfacing. Scale bar, 1 cm.

3. Lack of Quantitative Holographic Parameters:

The description of the holographic projection system in Figure 3a is overly qualitative. Key parameters necessary for evaluating display performance are missing, such as:

• The precise distance between the metasurface and the screen.

• The physical dimensions of the projected image on the screen.

• The field of view (FOV).

Without these metrics, it is impossible to assess the practical utility or the resolution limits of the proposed display.

OUR RESPONSE and **ACTIONS TAKEN**:

Following the reviewer's valuable suggestion, we incorporated additional quantitative descriptions of the holographic projection geometry in the revised manuscript and Supplementary Information.

Page 6, Main text:

"The device generates a holographically projected pixel array that serves as an interactive game field, spanning approximately 50 mm × 50 mm on a screen positioned 100 mm from the metadevice (Supplementary Note 3; Supplementary Fig. 12)."

Supplementary Note 3: Evaluation of the holographically projected pixel array

"Holographic projections from the organic metadevices were formed on a screen positioned 100 mm from the metadevice and recorded using a visible-light camera. The projected 36 holographic pixels span approximately 50 mm × 50 mm on the screen (Supplementary Fig. 12a). The outermost holographic pixels correspond to off-axis projection angles of approximately ±34° and ±33° along the horizontal and vertical directions, respectively, corresponding to an estimated field of view (FOV) of ~68° × 66°."

4. Insufficient Optical Characterization of AuNRs:

The optical characterization of the PANI-coated AuNRs is inadequate for a metasurface-focused study. Figure 1S alone does not provide sufficient data on the spectral response or the absolute intensity modulation during electrochemical cycling. The authors should also provide comprehensive extinction/scattering spectra. This is vital for evaluating the device's performance as a dynamic display.

OUR RESPONSE and **ACTIONS TAKEN**:

We thank the reviewer for this comment. As detailed in our responses to this reviewer above (Supplementary Fig. 6, page 21 of this response letter), we added the scattering spectra measured under different electrochemical states in the revised Supplementary Information.

5. Photothermal Stability and Operational Lifetime:

Given the emphasis on "stable operation" and the use of a laser source, the impact of photothermal effects cannot be ignored. Gold nanorods are efficient light-to-heat converters; under continuous laser illumination, local heating may accelerate the degradation of the PANI coating or the electrolyte. The authors must provide lifetime testing data, specifically plotting contrast/intensity loss vs. cycle count. A stability test under actual operating conditions (laser-illuminated cycling) is necessary to support the claim of a "stable" interactive system.

OUR RESPONSE and **ACTIONS TAKEN**:

We thank the reviewer for this comment. As detailed in our responses to this reviewer above (Supplementary Fig. 16, page 23 of this response letter), we performed long-term stability measurements of the metadevice under continuous laser illumination during electrochemical switching, corresponding to the practical operating conditions used for holographic projection and interactive operation.

Is the methodology sound? Does the work meet the expected standards in your field?

Not entirely. As noted in my previous comments, the methodology requires substantial upgrades to meet the field's standards. Specifically, the manuscript lacks quantitative assessments of pixel-level electrochemical crosstalk and rigorous stability testing under laser-induced photothermal conditions

OUR RESPONSE and **ACTIONS TAKEN**:

We thank the reviewer for this constructive comment. As detailed in our responses above to this reviewer, we added quantitative evaluations of pixel-level electrical crosstalk by sequentially addressing individual pixels while monitoring the intensity variations across the array, confirming negligible crosstalk in the independently addressable metadevice architecture (Supplementary Fig. 13, pages 21-22 of this response letter). In addition, long-term stability measurements were performed under continuous laser illumination during electrochemical switching (Supplementary Fig. 16, page 23 of this response letter).

Is there enough detail provided in the methods for the work to be reproduced?

No. The current "Methods" section lacks critical details necessary for reproduction, particularly regarding the precise localized deposition of AuNRs on pixel electrodes and the physical integration of the electrochemical cell within the 2D array. More transparent fabrication protocols and optical setup parameters are required.

OUR RESPONSE and **ACTIONS TAKEN**:

We thank the reviewer for this comment. As detailed in our responses to this reviewer above (Supplementary Fig. 1, page 24 of this response letter), we substantially expanded the methodological descriptions in the revised manuscript and Supplementary Information, including step-by-step fabrication procedures, localized PANI deposition, electrochemical device packaging, and holographic projection geometry. These additions provide detailed experimental information for reproducing the fabrication, electrochemical operation, and optical characterization of the metadevice platform.

Reviewer #1 (Remarks to the Author):

The authors have thoroughly revised their manuscript and addressed the previous comments point by point. The revised version is of higher quality and easier to understand. The revised manuscript has provided substantially expanded data to demonstrate both the operational stability and encapsulation feasibility of the platform. The issues related to resolution and pixel level validation have been addressed through the additional proof of concept demonstrations, accompanied by clearer and more physically grounded interpretations. Moreover, the authors have systematically investigated the effect of the PANI thickness on the switching performance and have included electrochemical data to characterize the evolution of the PANI properties.

In addition, the revision has incorporated quantitative evaluations of the key engineering figures of merit, including the diffraction efficiency, power consumption, cycling lifetime, and long-term operational drift. To resolve concerns regarding the orientation uniformity and phase gradient fidelity, statistical analyses based on the SEM images of the metasurface have been introduced. Furthermore, the rationale for employing circularly polarized light has been clarified, and the corresponding optical efficiency has been experimentally quantified. A quantitative characterization of electrical crosstalk has also been added, supported by new measurements and detailed analysis.

Overall, the revision has resolved the substantive concerns and resulted in a coherent, technically sound article with clear takeaways for readers. I recommend acceptance in its current form.

We thank the reviewer for fully supporting the publication of our work.

Reviewer #2 (Remarks to the Author):

The authors have answered my questions.

We thank the reviewer for supporting the publication of our work.

Reviewer #3 (Remarks to the Author):

I thank the authors for their careful revisions and detailed responses to my comments. I am generally satisfied with the changes made in the revised manuscript.

First, I appreciate the authors' efforts to clarify the novelty and positioning of the work relative to the related studies I suggested. I agree with the statement that "Neither work focused on programmable metasurface optics, holographic wavefront reconstruction, or interactive metadevice operation in the visible regime." The comparison with the prior literature helps to clearly establish the fundamental distinctions and contributions of the present study.

Second, regarding my concern about the limited theoretical analysis in the original submission, the authors have significantly strengthened the manuscript by providing detailed numerical investigations of the electrochemical–optical coupling mechanisms. The additional simulations and analyses relating to the optical scattering response to the geometry evolution offer valuable physical insight into the device operation.

Furthermore, I appreciate the authors' additional quantitative characterizations addressing my concerns regarding the claims of "negligible electrical crosstalk" and "stable operation." The new experimental evidence adequately supports these statements and improves the overall rigor of the work.

Finally, the concerns I raised regarding the electrochemical setup have also been satisfactorily addressed through the newly added experimental images and corresponding discussions.

Overall, the authors have carefully addressed my major comments, and the revised manuscript has been substantially improved. I have no further major concerns. I only have two minor suggestions that may further improve the clarity and reproducibility of the work:

We thank the reviewer for the positive assessment of the revised manuscript and for the constructive suggestions provided below.

1. In Supplementary Fig. 1b(iv), I am still curious about the identity of the filament-like material observed between adjacent pixels. It would be helpful if the authors could clarify whether this feature corresponds to residual resist, PANI, or another material. Such information may provide additional insight into the protective role of PMMA during the electrochemical processes and could be of interest to readers.

OUR RESPONSE and **ACTIONS TAKEN**:
In response, we added a magnified optical microscopy image of an individual metasurface pixel in Supplementary Fig. 1c, which shows that the filament-like features are confined to the boundaries of the PMMA partition masks introduced during fabrication step (iii) and remain visible after the electrochemical polymerization step (iv). Their spatial localization therefore supports their assignment as residual PMMA mask features. Furthermore, the negligible electrical crosstalk observed experimentally (Supplementary Fig. 13) confirms that these features do not form conductive pathways between adjacent pixels. We have added a corresponding clarification to the caption of Supplementary Fig. 1.

Supplementary Fig. 1:

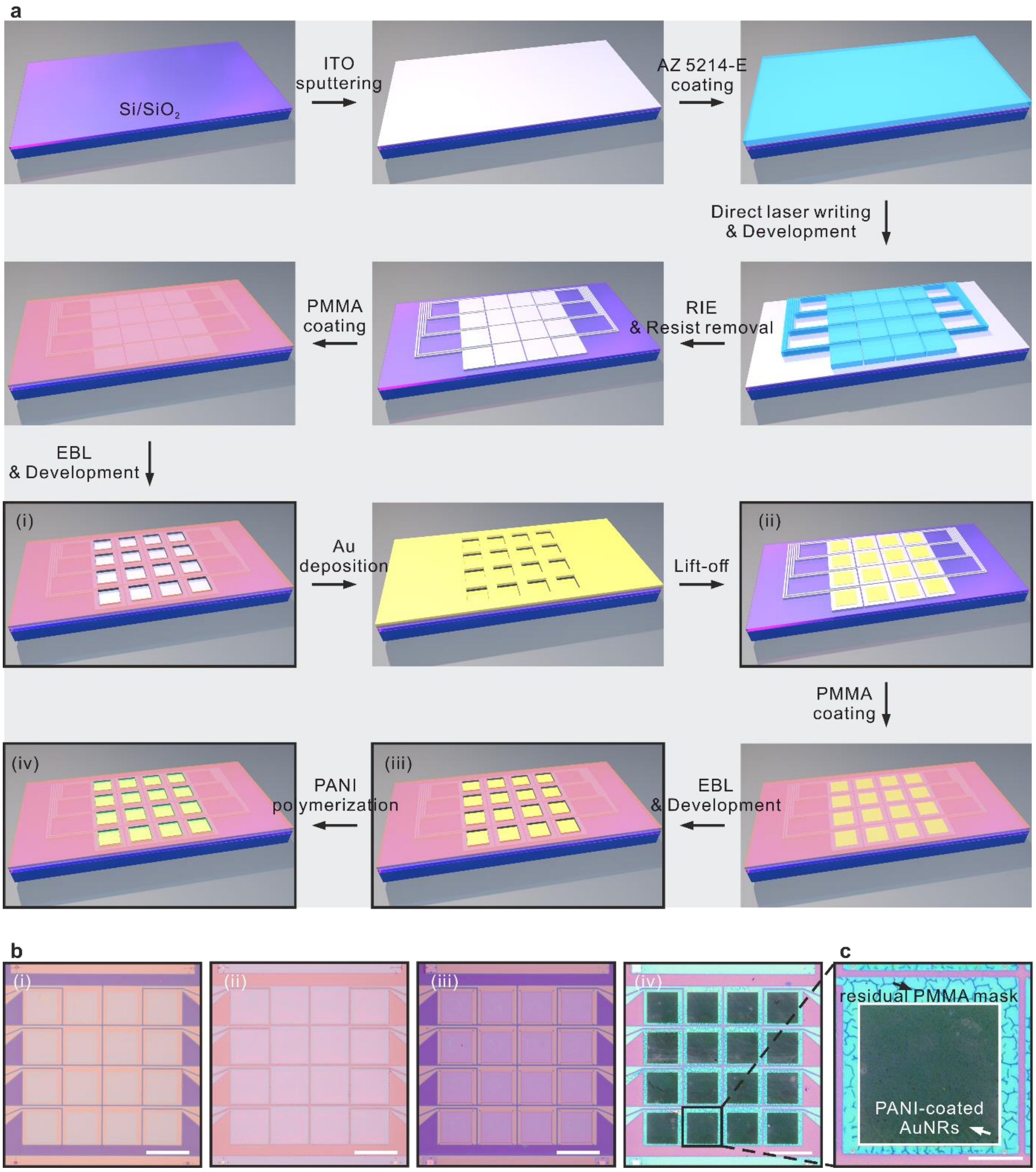

Supplementary Fig. 1 | Fabrication process of interactively addressable organic metadevices. a, Schematic illustration of the step-by-step fabrication process. **b**, Optical microscopy images of the metasurface array at fabrication steps (i)–(iv) shown in (**a**). Scale bar, 350 µm. **c**, Magnified optical microscopy image of one metasurface pixel. Scale bar, 100 µm.

2. For the holographic pixel setup, I would recommend adding a simple schematic illustration with clearly labeled dimensions and geometric parameters. While the current description is understandable, a dedicated schematic would allow readers to more intuitively visualize the device architecture and experimental configuration, thereby improving clarity and facilitating reproducibility.

OUR RESPONSE and **ACTIONS TAKEN**:
In response, we added a new schematic illustration of the holographic projection setup in Supplementary Fig. 12a, with the key geometric parameters and dimensions explicitly labeled.

Supplementary Fig. 12:

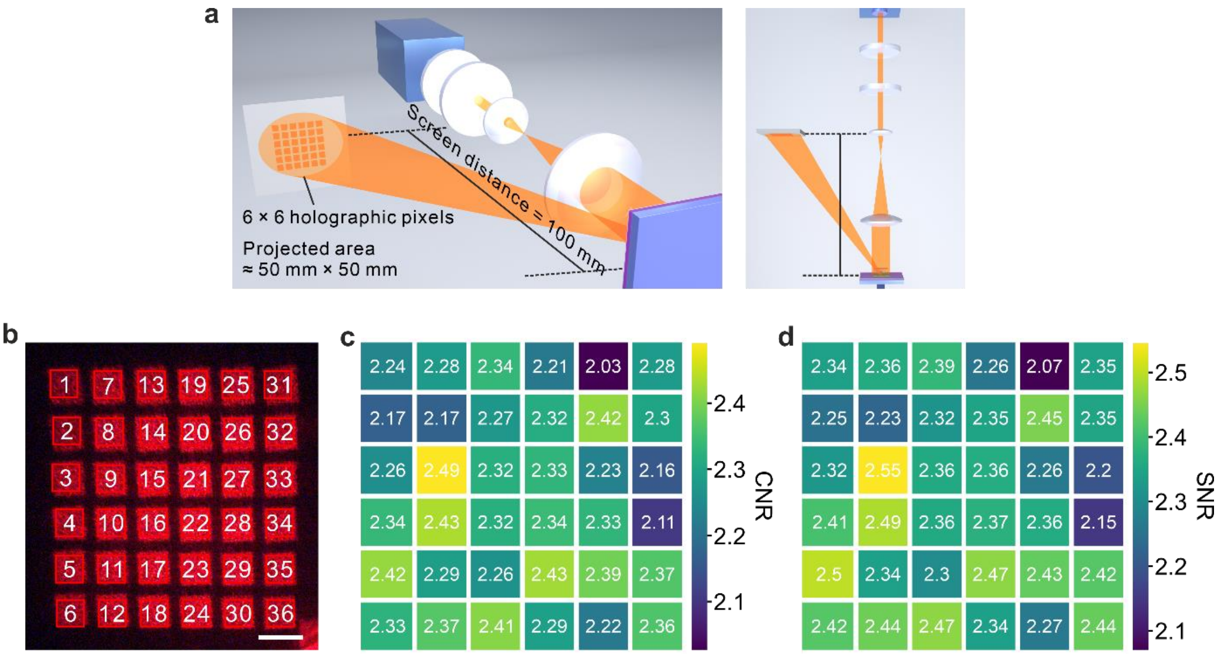

Supplementary Fig. 12 | Evaluation of the holographically projected pixel array. a, Schematic illustration of the optical measurement setup for the 6 × 6 pixel array. **b**, Representative holographic projection of the 6 × 6 pixel array on the screen. The hologram region of each pixel is marked by a red square with the corresponding pixel index used for analysis. Scale bar, 10 mm. **c**, Spatial maps of pixel-wise CNR and **d**, SNR across the 6 × 6 metasurface pixel array, extracted from the hologram regions and the dark background in (**b**) and averaged over 5 holographic projection images.
